# Identifying Modifiable Factors and Their Joint Effect on Amyotrophic Lateral Sclerosis: A Prospective Cohort Study in the UK Biobank

**DOI:** 10.1002/brb3.71771

**Published:** 2026-09-23

**Authors:** Xinyu Yang, Jing Yuan, Sen Huang, Xiaoli Yao

**Affiliations:** ^1^ Department of Neurology The First Affiliated Hospital Sun Yat‐sen University Guangzhou China; ^2^ Guangdong Provincial Key Laboratory of Diagnosis and Treatment of Major Neurological Diseases The First Affiliated Hospital Sun Yat‐sen University Guangzhou China; ^3^ National Key Clinical Department and Key Discipline of Neurology The First Affiliated Hospital Sun Yat‐sen University Guangzhou China

**Keywords:** amyotrophic lateral sclerosis, exposome‐wide association study, modifiable factors

## Abstract

**Background:**

Hypothesis‐driven studies have linked numerous risk factors to amyotrophic lateral sclerosis (ALS). However, the full extent of their diversity and co‐occurrence has not been adequately appreciated. This study aims to comprehensively explore the modifiable risk factors for ALS and to evaluate the potential benefit of controlling these factors.

**Methods:**

This prospective cohort study utilized data from UK Biobank participants enrolled between 2006 and 2010 for whom complete data were available. An exposome‐wide association study (EWAS) was employed to screen for modifiable risk factors, adjusted for sex and age. The main outcome was the first recorded diagnosis of ALS. The associations of modifiable risk factors with ALS risk were assessed using hazard ratios and 95% confidence intervals. The population attributable fraction (PAF) was used to estimate the joint effects of modifiable factors.

**Results:**

This study included a total of 326,050 participants and assessed 184 modifiable risk factors. During follow‐up, 744 participants were diagnosed with ALS for the first time. EWAS, adjusted for sex and age, identified 45 modifiable factors associated with ALS incidence across six domains (excluding early life), including varicella, lower education, and peak expiratory flow. Analysis using the PAF indicated that 25.94%–87.40% of ALS cases could potentially be prevented.

**Conclusions:**

In this study, newly identified risk factors for ALS through the EWAS may serve as targets for future studies. Interventions targeting modifiable risk factors, particularly those related to lifestyle and socioeconomic status, may help reduce the incidence of ALS.

## Graphical abstract

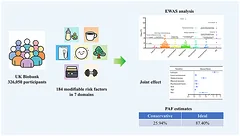

**Abbreviations**
ALS: amyotrophic lateral sclerosis
CI: confidence interval
EWAS: exposome‐wide association study
HR: hazard ratio
ICD: international classification of diseases
PAF: population attributable fraction
SD: standard deviation.
SES: socioeconomic status

## 1 Introduction

Amyotrophic lateral sclerosis (ALS) is a fatal neurodegenerative disorder driven by the progressive demise of motor neurons (Feldman et al. 2022). With no curative treatments available, targeting modifiable risk factors for prevention may be the most important—and perhaps the only—practical means of reducing the ALS burden (Yang et al. 2025). The hypothesis‐driven approach has historically been widely used to investigate risk factors for ALS and underpin preventive efforts (Zhu et al. 2025; Hu et al. 2025; Vaage et al. 2024). However, these approaches have certain limitations. First, owing to the interdependence among risk factors, isolated exposure analyses risk overestimating effect sizes and inflating the false‐positive rate (Gao et al. 2025). Second, these studies are prone to selective reporting, which limits research reproducibility (Patel and Ioannidis 2014). Third, examining risk factors individually or in small sets overlooks the synergistic effects of combined exposures (Al‐Chalabi and Hardiman 2013). Finally, overall contribution of risk factors to ALS remains unclear from single‐exposure analyses. Nevertheless, growing evidence now supports the effectiveness of multidomain lifestyle interventions for ALS prevention (Westeneng et al. 2021; Korner et al. 2019).

The exposome‐wide association study (EWAS) is an unbiased, hypothesis‐free strategy for systematically interrogating associations between multiple variables and a health outcome. Its utility has been demonstrated in complex conditions, including dementia, depression, and diabetes (Zhang et al. 2023; Choi et al. 2020; Sheehan et al. 2020). By examining multiple exposures simultaneously, EWAS can validate established risk factors with reduced bias and false‐positives, while also enabling the discovery of novel ones (Lin et al. 2022; Manrai et al. 2016). Analogous to genome‐wide association studies (GWAS), EWAS adopts standardized analytical workflows, producing findings that are more robust than those derived from hypothesis‐guided investigations (Manrai et al. 2016). Furthermore, by constructing composite scores (Zhang et al. 2023) and calculating the population attributable fraction (PAF) (Ritchie et al. 2010), the joint effects of multiple risk factors can be quantified, along with their respective contributions to ALS prevalence.

**FIGURE 1:**
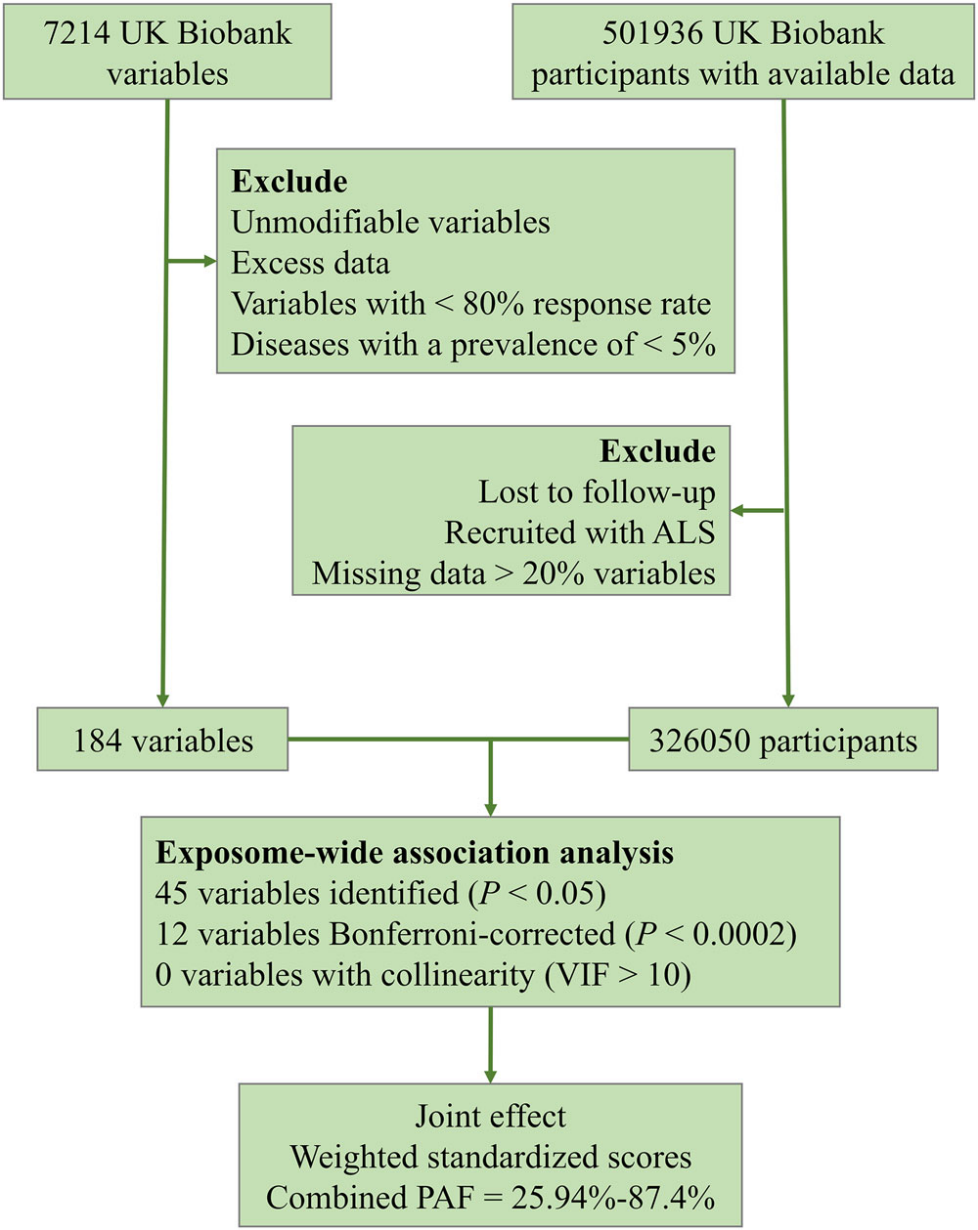
Analytical procedure to identify modifiable risk factors associated with incident ALS in the UK Biobank. ALS, amyotrophic lateral sclerosis; VIF, variance inflation factor.

In this study, we conducted an EWAS using phenotypic data from 326,050 UK Biobank participants to systematically identify risk factors for ALS. We then constructed composite scores for each domain to evaluate the joint effects of multidomain exposures. Finally, we estimated PAFs for individual domains and for all factors combined so as to assess the potential impact of preventive interventions (Figure 1).

## 2 Methods

### 2.1 Study Design

First, we conducted an EWAS using Cox proportional hazards models to examine 184 modifiable risk factors in relation to incident ALS. We then stratified associated factors by age and sex, evaluated joint effects by constructing domain‐specific weighted standardized scores, and assessed their independent contributions in a multivariable model. Finally, we estimated PAFs while accounting for concurrent risks across domains within the same individual.

### 2.2 Study Population and ALS Diagnosis

This study used data from the UK Biobank, which recruited 501,936 participants between 2006 and 2010 (Bycroft et al. 2018). Participants ranged in age from 40 to 69 years and represented diverse occupational backgrounds. At baseline, participants attended the research center and, under the guidance of research personnel, completed questionnaires (either paper‐based or electronic) regarding lifestyle factors, environmental exposures, and prior medical history. Throughout the follow‐up phase, the UK Biobank conducted participant surveillance via web‐based questionnaires and linkage to national healthcare registries. To exclude reverse causation, ALS patients who developed the disease within 6 years after baseline were not included in the analysis (Zhang et al. 2023). Participants were followed from baseline until the earliest of ALS diagnosis, loss to follow‐up, or the end of follow‐up (October 2025). ALS cases were identified using International Classification of Diseases (ICD) codes, with G12.2 denoting ALS (Pang et al. 2025).

### 2.3 Modifiable Factors

Variables with >20% missingness and those considered non‐modifiable were excluded. The final analytical dataset comprised 184 modifiable factors assessed at baseline (Table S1), grouped into seven broad categories as follows: early life factors, lifestyle behaviors, local environment, medical history, physical measures, psychosocial factors, and socioeconomic status (SES) (Zhang et al. 2023). Multiple‐choice categorical variables were dichotomized into binary variables. For instance, the type of vitamins consumed was recoded as a binary variable indicating whether a specific vitamin was consumed. Variables with only one category were excluded. We further imposed a minimum threshold of more than 500 cases for inclusion in the analysis. For diseases, we extracted data from the “First occurrence” category, which was coded using ICD‐10 codes, with the date of first diagnosis documented. Diseases with a prevalence rate below 2% were also excluded. For continuous variables, they were divided into tertiles after imputation (Table S2). With the exception of computer usage time, for which the middle tertile was used as the reference category, the lower tertile was selected as the reference for all other variables. For the factors retained after the EWAS analysis, the generalized variance inflation factor (GVIF) and its adjusted values were employed to test for multicollinearity among them.

### 2.4 Statistical Analyses

Missing values were handled by multiple imputation (5 datasets, 10 iterations), with pooled estimates used as the final imputed data. Cox proportional hazards regression was applied to perform the EWAS and to estimate the hazard ratios for each baseline exposure in relation to incident ALS. Statistical significance was evaluated using *Z*‐tests, from which *Z* statistics and two‐sided *P* values were derived. Age, sex, and genetic mutations are recognized as confounding factors closely associated with ALS (Feldman et al. 2022). The pathogenic genes of ALS and their corresponding rsIDs were retrieved from the ClinVar database of the National Centre for Biotechnology Information (Udine et al. 2023). Given the large number of ALS‐associated pathogenic genes and their low mutation rates, only those mutations with definitive pathogenicity were included in the present study (Table S3). However, univariate Cox proportional hazards analysis did not identify genetic mutations as a risk factor for ALS (Table S4). Therefore, only age and sex were included as covariates in the subsequent analyses.

The EWAS‐significant variables were classified into six predefined domains, namely, lifestyle, local environment, medical history, physical measures, psychosocial factors, and SES. No risk factors associated with ALS were found in the early‐life domain; therefore, this domain was excluded from subsequent analyses. For directional consistency, protective exposures (hazard ratio [HR] < 1) were inverted, whereas harmful exposures (HR > 1) were assigned a unit contribution to the corresponding domain score. Weighted standardized scores for each domain were then computed using *β* coefficients derived from Cox proportional hazards models, with all constituent risk factors adjusted for one another in addition to age and sex (Lourida et al. 2019). A domain‐specific score was constructed by weighting each binary variable by its *β* coefficient, summing the weighted values, and dividing by the sum of all coefficients in that domain, whereby higher scores corresponded to increased risk factor exposure.

As an estimate of the disease burden that could be avoided by reducing or removing a given risk factor, PAF is a cornerstone metric for informing public health decisions and priority‐setting in policy programs. In the present study, we converted unfavorable characteristics across the six domains into intermediate ones to generate more conservative estimates. We additionally transformed both unfavorable and intermediate characteristics into favorable ones to derive the theoretical ideal PAF values. To account for the nonindependence across the six domains, communality was estimated via principal component analysis and applied as a weighting factor for each domain‐specific PAF, facilitating the derivation of both an aggregate weighted PAF and domain‐specific weighted PAF (Livingston et al. 2020). This method addresses the concurrent presence of risk factors within individuals, thereby preventing the inflation of PAF estimates attributable to risk factor interactions.

All *P* values were two‐sided, and all analyses were performed using R within the UK Biobank research analysis platform. The threshold for Schoenfeld's residuals was set at *P* < 0.0001 (Zhang et al. 2023; Ganna and Ingelsson 2015).

## 3 Results

### 3.1 Identification of ALS Risk Factors in Prospective EWAS

**FIGURE 2:**
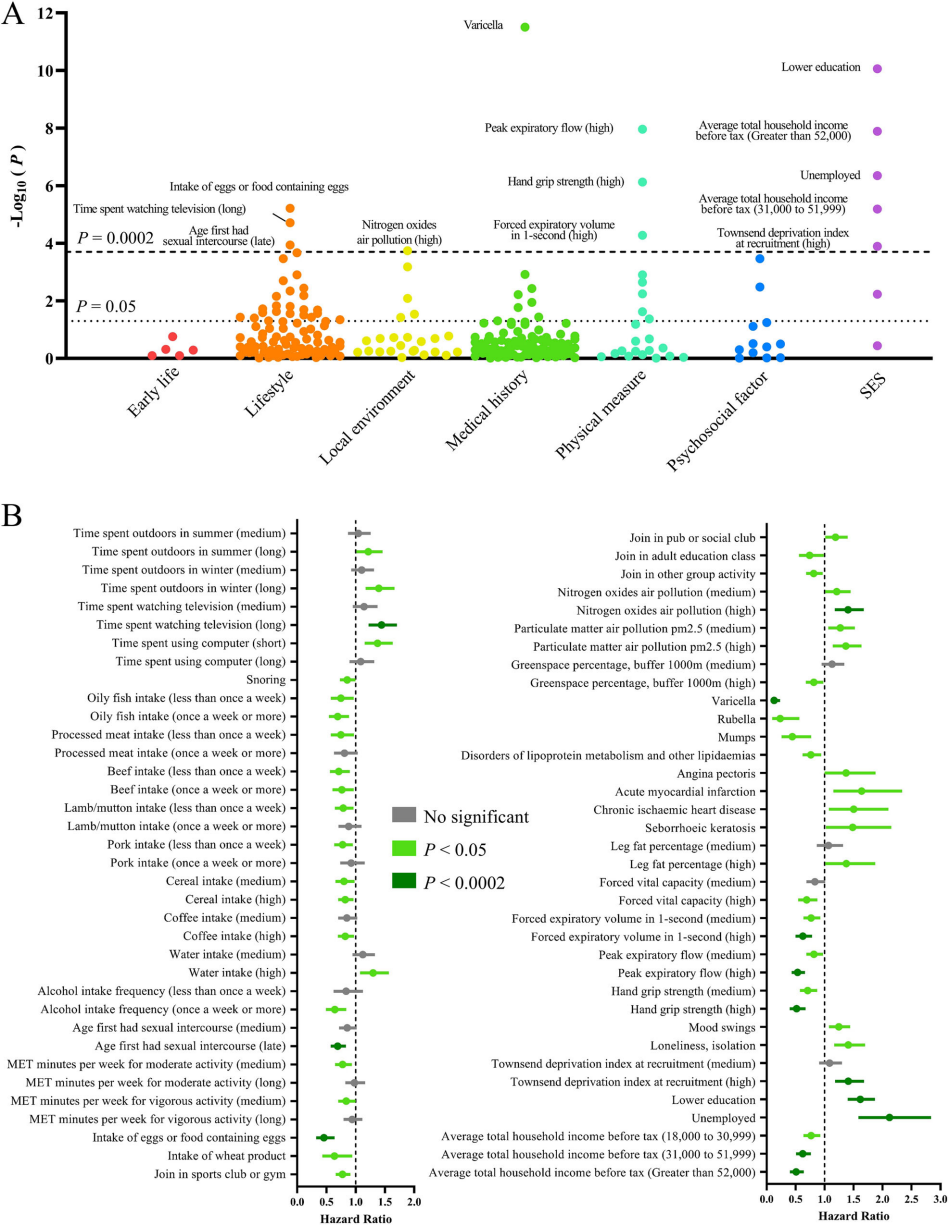
Associations between modifiable risk factors and incident ALS. (A) The *x* axis shows the category domains and the *y* axis represents statistical significance (−log10 of the *P* value). (B) Dots represent hazard ratios, and horizontal lines indicate corresponding 95% CIs. Hazard ratios were calculated using Cox proportional hazards regression analysis after adjustments for age at baseline and sex. SES, socioeconomic status.

This study included 326,050 individuals with an average age of 55.89 years, of whom 744 participants developed ALS after recruitment (Table 1). Of the 184 risk factors considered, 45 were significantly associated with ALS (Figure 2 and Table S5). Multicollinearity analysis of the 45 variables revealed no evidence of collinearity (Table S6). Among them, 27 factors showed potentially protective effects and 18 were detrimental. A total of 12 variables continued to exhibit a significant association with ALS after Bonferroni correction. Among the top 10 factors, 7 reduced liability for ALS: varicella (HR = 0.13, 95% confidence interval [CI] = 0.07–0.23, *P* = 3.12 × 10^−12^), peak expiratory flow (high: HR = 0.53, 95% CI = 0.43–0.66, *P* = 1.10 × 10^−8^), average total household income before tax (greater than 52,000: HR = 0.51, 95% CI = 0.40–0.64, *P* = 1.30 × 10^−8^; 31,000–51,999: HR = 0.62, 95% CI = 0.51–0.77, *P* = 6.5 × 10^−8^), handgrip strength (high: HR = 0.52, 95% CI = 0.40–0.67, *P* = 7.44 × 10^−7^), intake of eggs or food containing eggs (high: HR = 0.46, 95% CI = 0.33–0.64, *P* = 6.13 × 10^−6^), forced expiratory volume in 1‐second (high: HR = 0.63, 95% CI = 0.50–0.79, *P* = 5.32 × 10^−5^), age first had sexual intercourse (late: HR = 0.69, 95% CI = 0.57–0.83, *P* = 1.16 × 10^−4^); and 3 had increased liability for ALS: lower education (HR = 1.62, 95% CI = 1.40–1.87, *P* = 8.68 × 10^−11^), unemployment (HR = 2.12, 95% CI = 1.58–2.84, *P* = 4.46 × 10^−7^), time spent watching television (long: HR = 1.44, 95% CI = 1.22–1.71, *P* = 1.95 × 10^−5^).

**TABLE 1:** Baseline characteristics of studied population.

Characters	Label	Number or mean	Percentage or SD
Population size	Max	326,050	
Time	Days	4558.91	1232.95
Age	Years old	55.89	7.87
Follow‐up ALS	Yes	744	0.23%
	No	325,306	99.77%
Sex	Male	146,135	44.82%
	Female	179,915	55.18%
Gene mutation	Yes	18,927	5.8%
	No	307,123	94.2%

Abbreviation: SD, standard deviation.

### 3.2 Risk Factors in ALS Subgroups

**FIGURE 3:**
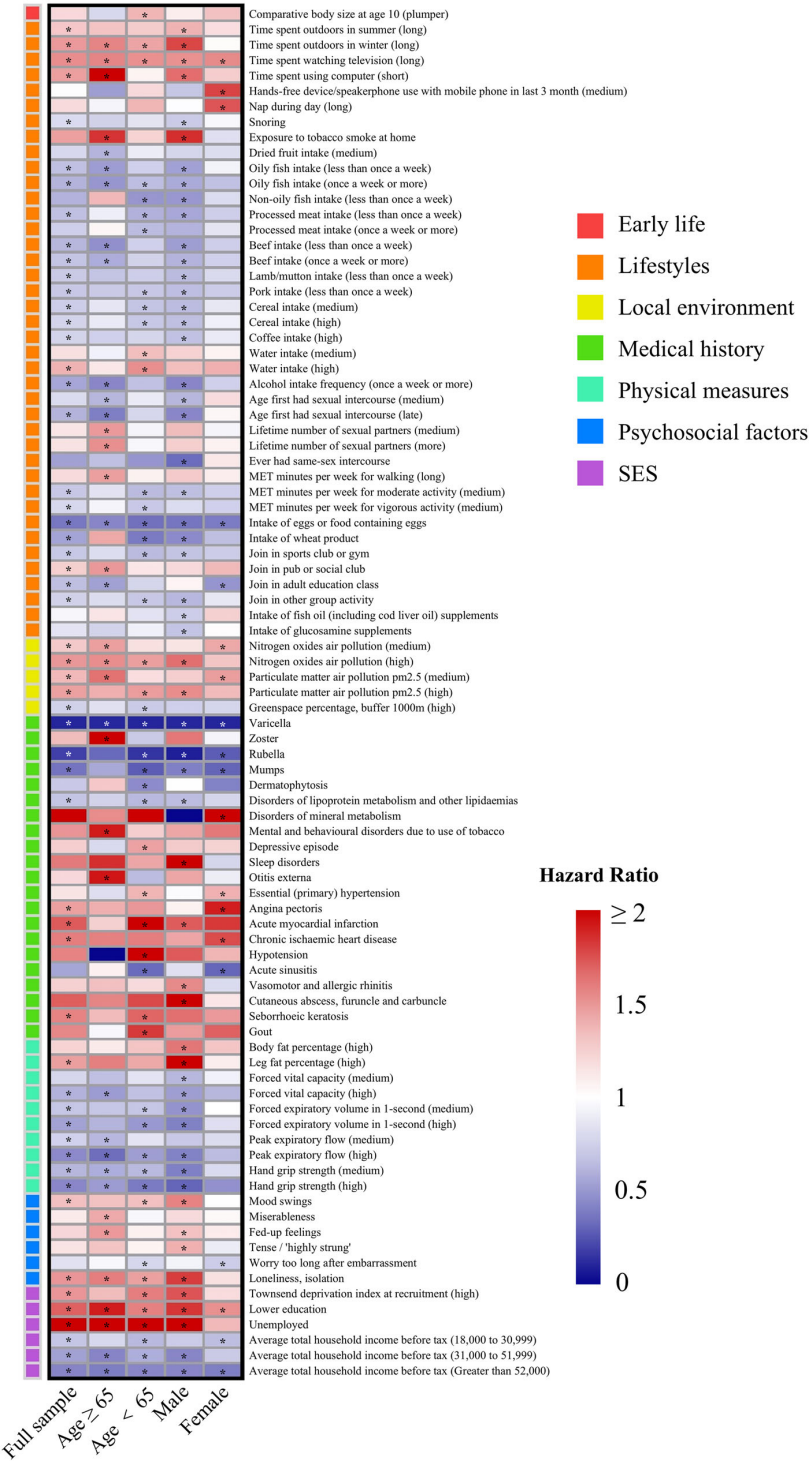
Summary heat map for significant factors in EWAS analysis across the full sample and subgroups. Models were adjusted for baseline age and sex. The color of cells indicates the effect sizes (HR) between each risk factor and incident ALS. Asterisks in cells represent significant associations (*P *< 0.05). SES, socioeconomic status.

It is well established that sex and age are associated with ALS. Figure 3 and Table S7 report the EWAS results in subgroups. Comparable association patterns were evident upon stratification of the results by age and sex. In the subgroup aged ≥ 65 years (Figure S1), the top three factors were time spent using computer (short: HR = 2.25, 95% CI = 1.67–3.05, *P* = 1.41 × 10^−7^), lower education (HR = 1.88, 95% CI = 1.47–2.42, *P* = 6.58 × 10^−7^), and peak expiratory flow (high: HR = 0.42, 95% CI = 0.29–0.61, *P* = 3.72 × 10^−6^). In the subgroup aged < 65 years (Figure S2), the top three factors were varicella (HR = 0.13, 95% CI = 0.07–0.25, *P* = 1.34 × 10^−9^), unemployment (HR = 2.09, 95% CI = 1.54–2.83, *P* = 2.36 × 10^−6^), and average total household income before tax (greater than 52,000: HR = 0.52, 95% CI = 0.39–0.68, *P* = 3.37 × 10^−6^). In males (Figure S3), the top three factors were unemployment (HR = 3.09, 95% CI = 2.16–4.40, *P* = 5.05 × 10^−10^), lower education (HR = 1.79, 95% CI = 1.47–2.17, *P* = 6.45 × 10^−9^), and handgrip strength (high: HR = 0.38, 95% CI = 0.27–0.53, *P* = 1.76 × 10^−8^). In females (Figure S4), the top three factors were varicella (HR = 0.13, 95% CI = 0.05–0.31, *P* = 5.06 × 10^−6^), average total household income before tax (greater than 52,000: HR = 0.48, 95% CI = 0.33–0.71, *P* = 1.85 × 10^−4^), and disorders of mineral metabolism (HR = 5.37, 95% CI = 2.00–14.38, *P* = 8.39 × 10^−4^). The early life factor was significant only in the subgroup aged < 65 years.

### 3.3 Joint Effects of Identified Factors on ALS

**FIGURE 4:**
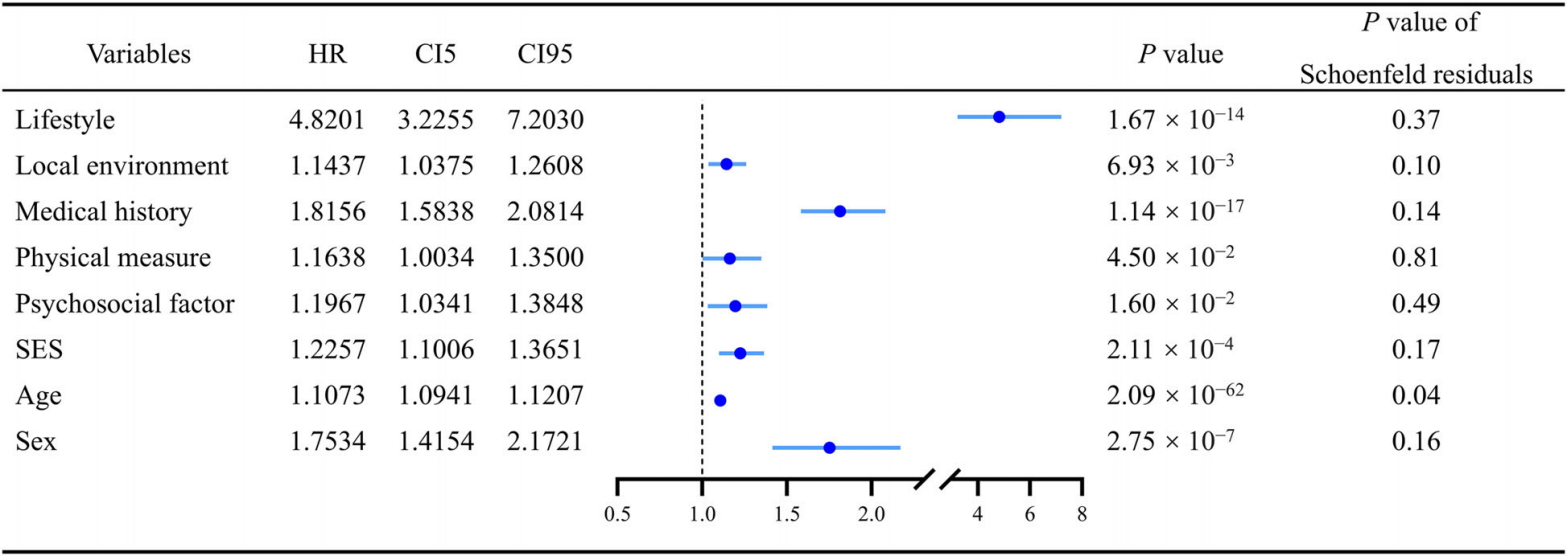
Associations between six domains and ALS. The associations were estimated by applying Cox model, including all six domains, after adjustment for age and sex. Dots represent hazard ratios. Horizontal lines indicate corresponding 95% CIs. CIs, confidence intervals; HR, hazard ratio; SES, socioeconomic status.

Following standardized scoring of all participants, the multivariable Cox model was employed to evaluate the joint effects of the six domains on ALS (Figure 4). All six domains increased the risk of ALS: lifestyle (HR = 4.82, 95% CI = 3.23–7.20, *P* = 1.67 × 10^−14^), local environment (HR = 1.14, 95% CI = 1.04–1.26, *P* = 6.93 × 10^−3^), medical history (HR = 1.82, 95% CI = 1.58–2.08, *P* = 1.14 × 10^−17^), physical measure (HR = 1.16, 95% CI = 1.00–1.35, *P* = 4.50 × 10^−2^), psychosocial factor (HR = 1.20, 95% CI = 1.03–1.38, *P* = 1.60 × 10^−2^), and SES (HR = 1.23, 95% CI = 1.10–1.37, *P* = 2.11 × 10^−4^).

### 3.4 PAF Estimates for Preventing ALS Across the Six Domains

When unfavorable characteristics were converted to intermediate characteristics for all participants (conservative model), the PAF estimated that 25.94% of ALS cases could be prevented (Table 2); when all factors were shifted to favorable levels (ideal model), this proportion could be further increased to 87.40% (Table 3). Under the conservative model, SES was estimated to confer the greatest preventive benefit, with a 6.79% reduction in ALS incidence, followed by local environment (6.63%), psychosocial factors (5.99%), medical history (4.56%), physical measures (1.84%), and lifestyle (0.13%). Under the ideal model, lifestyle exhibited the largest preventive effect, corresponding to a 41.31% reduction in ALS incidence. Other domains were responsible for ALS cases as follows: medical history (21.25%), psychosocial factors (8.91%), SES (7.10%), local environment (5.62%), and physical measures (3.21%).

**TABLE 2:** Weighted and unweighted population attributable fraction (PAF) for the six domains in conservative model.

Variables	PE	Unweighted PAF	CI5	CI95	Communality	Weighted PAF
Lifestyle	0.0005	0.0018	0.0011	0.003	0.0835	0.0013
Local environment	0.7154	0.0932	0.0261	0.1572	0.0285	0.0663
Medical history	0.0839	0.0641	0.0467	0.0832	0.0004	0.0456
Physical measure	0.1626	0.0259	0.0005	0.0538	0.0328	0.0184
Psychosocial factor	0.4682	0.0843	0.0157	0.1527	0.0176	0.0599
SES	0.4679	0.0955	0.045	0.1459	0.734	0.0679
Overall weighted PAF						0.2594

*Note*: PAF expressed as decimals.

Abbreviations: CI, confidence interval; PAF, population attributable fraction; PE, prevalence of exposure; SES, socioeconomic status.

**TABLE 3:** Weighted and unweighted population attributable fraction (PAF) for the six domains in ideal model.

Variables	PE	Unweighted PAF	CI5	CI95	Communality	Weighted PAF
Lifestyle	0.8357	0.7615	0.6503	0.8383	0.0824	0.4131
Local environment	0.8044	0.1036	0.0293	0.1734	0.0308	0.0562
Medical history	0.7896	0.3917	0.3155	0.4606	0.0003	0.2125
Physical measure	0.3834	0.0591	0.0013	0.1183	0.0308	0.0321
Psychosocial factor	1.0000	0.1643	0.0330	0.2779	0.0153	0.0891
SES	0.6670	0.1309	0.0629	0.1958	0.7502	0.0710
Overall weighted PAF						0.8740

*Note*: PAF expressed as decimals.

Abbreviations: CI, confidence interval; PAF, population attributable fraction; PE, prevalence of exposure; SES, socioeconomic status.

## 4 Discussion

Given the multifactorial etiology of ALS, an EWAS was first employed to identify risk factors across six domains: lifestyle, local environment, medical history, physical measures, psychosocial factors, and SES. In line with the literature, our study identified that lower education (Pamphlett and Parkin Kullmann 2024), lower SES (Shojaie et al. 2024), exposure to tobacco (Westeneng et al. 2021; Zhan and Fang 2019), air pollution (Wu et al. 2023; Pedde et al. 2025; Saucier et al. 2025), vigorous physical activity (Visser et al. 2018; Chapman et al. 2023), and long time spent watching television (Rosenbohm et al. 2021) (indicative of physical inactivity) were risk factors for ALS. Our findings also indicate that moderate physical activity is associated with a lower incidence of ALS (Vaage et al. 2024; Gallo et al. 2016). However, vigorous physical activity is an adverse factor for ALS (Chapman et al. 2023; Bate et al. 2026). There appears to be a potential nonlinear or threshold relationship between physical activity volume/intensity and the degenerative processes of motor neurons. With regard to dietary factors, previous studies suggested that high‐energy diets and ketogenic diets are beneficial for ALS (Dorst et al. 2015; Herrmann et al. 2026; Xu et al. 2024; Wills et al. 2014). In line with this, our study similarly observed inverse associations between the consumption of eggs, fish, beef, lamb, pork, and cereals and the incidence of ALS. Besides, inverse associations with ALS risk have also been observed for alcohol consumption (Westeneng et al. 2021; Chiò and Swash 2015) and coffee intake (Pupillo et al. 2017). However, whether alcohol plays a protective or harmful role in ALS remains a matter of debate, and this controversy may arise from variations in the type of alcoholic beverage, frequency of drinking, and volume of intake across studies (Chiò and Swash 2015; Yu et al. 2020).

Previous studies demonstrated that good respiratory function (Plowman et al. 2023), strong handgrip strength (Klickovic et al. 2025), and lipidaemia (Thompson et al. 2021; Goldstein et al. 2008) were favorable prognostic factors for ALS, and that individuals with ALS exhibited obvious feelings of loneliness and mood swings (Consonni et al. 2024; van der Hulst et al. 2014). Our study also revealed that better respiratory function, stronger handgrip strength, and higher lipid levels were inversely associated with ALS risk, whereas feelings of loneliness and mood swings showed positive associations with the disease. To mitigate the potential impact of reverse causality, we set the follow‐up time cut‐off at more than 6 years after baseline (Zhang et al. 2023). Although decreased grip strength and reduced respiratory function can be early manifestations of ALS, they may also represent “motor reserve” (Bede et al. 2021). By analogy with cognitive reserve, which reduces dementia risk, greater motor reserve may similarly protect against ALS. Indeed, subclinical corticospinal tract alterations and spinal cord atrophy have been documented in unaffected relatives of ALS patients (Querin et al. 2019).

Several factors in this study have not been fully explored to date: varicella, rubella, mumps, later age at first sexual intercourse, and outdoor activity exposure, as well as adverse factors: acute myocardial infarction, chronic ischemic heart disease, seborrheic keratosis, high water intake, and long time spent outdoors in summer and winter. These factors lack clear biological rationales and require more cautious interpretation. This study found that some viral infections may exert a protective effect against the development of ALS, a protective effect that has also been observed in dementia, which may be related to immune modulation (Pomirchy et al. 2025). However, a Mendelian randomization study yielded no evidence of a causal relationship between human herpesviruses and ALS (Zheng et al. 2023). A longitudinal and cross sectional study on viral exposure and neurodegenerative diseases found that a history of influenza and pneumonia was significantly associated with ALS (Levine et al. 2023). The direct evidence linking viral exposure to ALS remains limited, and this warrants further investigation. A population‐based study on heart disease and ALS reported that total cardiovascular disease, ischemic heart disease, hypertensive disease, and atherosclerosis were adverse risk factors for ALS. However, no further subclassification of heart disease was performed (Kioumourtzoglou et al. 2016). In particular, outdoor activity in the UK Biobank is frequently linked to outdoor physical exercise (Kröger et al. 2025; Miguet et al. 2021). Higher water intake and long time spent outdoors were found to have adverse effects on the onset of ALS, which may attribute to the greater amount of physical activity performed outdoors. The associations of age at first sexual intercourse, the outdoors, and seborrheic keratosis with ALS risk have not been examined in any studies to date.

In the conservative model, SES, local environmental, and psychological factors represented the key contributors to the PAF of ALS, with respective contributions of 6.79%, 6.63%, and 4.56%. Nevertheless, their preventive effects were relatively limited. In the ideal model, lifestyle had the largest potential, with a 41.31% reduction in ALS incidence, whereas medical history accounted for 21.25%. One study demonstrated that identification of modifiable lifestyle risk factors provided an opportunity to reduce the risk of developing ALS (Westeneng et al. 2021). These findings suggest that lifestyle‐related and comorbidity‐related factors are inversely associated with ALS risk, highlighting potential avenues for future preventive research. However, it is worth noting that the figure of 87.4% of ALS cases being preventable through modifiable risk factors in the ideal model seems excessively high. First, this 87.4% is derived from a mathematical calculation that aggregates risks across six major domains (rather than a single risk factor) under highly idealized conditions, which makes this number extremely elevated. Second, 87.4% represents a theoretical upper limit of prevention under a statistical model, rather than a realistically achievable target. Therefore, we do not believe that 87.4% means we can definitely prevent more than 80% of ALS cases; rather, it demonstrates that the risk of ALS can be influenced by large‐scale, multidomain, and long‐term interventions. ALS may have greater preventive potential than we previously thought.

It should be noted that some of the identified modifiable factors represent promising targets for individual level risk management, warranting further investigation in interventional settings. These include lifestyle, medical history, psychosocial factor, and physical measure. Although multiple pathogenic genes have been associated with ALS, no gene mutations were identified as a risk factor in the present cohort. This observation may be attributed to the incomplete penetrance of pathogenic genes (Turner et al. 2013). Furthermore, it also confirms that ALS is a multifactorial outcome involving genes, environment, and time, particularly in non‐familial ALS patients (Feldman et al. 2022). Nonetheless, local environmental determinants and socioeconomic variables, given their population‐level nature, are potentially responsive to community‐scale modifications. This observation underscores an urgent call for governmental and institutional action, with a particular focus on low and middle income countries, to advance a combined agenda of pollution abatement, ecological preservation, employment generation, and educational equity.

This study benefits from several notable advantages, including its substantial sample size, broad coverage of exposure variables, and the reliable data infrastructure of the UK Biobank, which together enabled a thorough examination of modifiable determinants. In addition, we undertook an EWAS of unprecedented scale, aimed at elucidating the combined effects of multiple exposures. These methodological attributes lend credence to our contributions toward the prevention of ALS, particularly within the framework of big data epidemiology.

A number of methodological constraints should be acknowledged. Foremost, the potential for volunteer bias may compromise the external validity of our observations. Moreover, the participants' medical histories were derived from a variety of sources, with some relying on patient self‐reports, which may have introduced bias. Furthermore, the generalizability of our PAF estimates to other settings is limited, as these estimates are inherently tied to the specific epidemiological characteristics of the source population. PAF as a preventable proportion depends on assumptions of causality, adequate control of confounding, and the actual modifiability of the included exposures. Given that PAF assumes causality—a premise our observational EWAS may not confirm—these estimates should be interpreted cautiously. Additionally, the lack of electromyography and ALSFRS‐R measurements within the UK Biobank precluded a more nuanced clinical assessment. Lastly, although we implemented rigorous statistical adjustments—including collinearity diagnostics and covariate control for sex and age—the possibility of residual confounding remains. Accordingly, we advocate for future studies employing hypothesis‐driven designs, including randomized controlled trials or prospective cohorts with prolonged surveillance and extensive covariate adjustment, to verify the true effect sizes.

## 5 Conclusions

In conclusion, this prospective study indicates that deterioration in lifestyle, local environment, medical history, physical measure, psychological factor, and SES is associated with an increased risk of ALS. The estimation of PAF in this study highlights the importance of individual interventions on lifestyle. Against the backdrop of global aging, the formulation of public health policies should attach importance to the SES and local environment of the population.

## Author Contributions

**Xinyu Yang**: writing – original draft, formal analysis. **Jing Yuan**: writing – review and editing, visualization, project administration. **Sen Huang**: conceptualization, methodology. **Xiaoli Yao**: conceptualization, data curation, supervision, funding acquisition. All authors have read and agreed to the published version of the manuscript.

## Funding

This study was supported by grants from the National Natural Science Foundation of China (82271448), Guangdong Provincial Clinical Research Centre for Neurological Diseases (2020B1111170002), Guangdong Province International Cooperation Base for Early Intervention and Functional Rehabilitation of Neurological Diseases (2020A0505020004), Guangzhou Major Difficult and Rare Diseases Project (2024MDRD02), Guangdong Provincial Engineering Centre for Major Neurological Disease Treatment, Guangdong Provincial Translational Medicine Innovation Platform for Diagnosis and Treatment of Major Neurological Disease, Guangzhou Clinical Research and Translational Centre for Major Neurological Diseases.

## Ethics Statements

The protocol of the UK Biobank study was conducted in accordance with the Declaration of Helsinki. This study was approved by the North West Multi‐Centre Research Ethics Committee (reference 11/NW/0382, 16/NW/0274, and 21/NW/0157).

## Conflicts of Interest

The authors declare no conflicts of interest.

## Supporting information




**Supplementary Material**: brb371771‐sup‐0001‐SuppMat.docx

## Data Availability

The dataset supporting the conclusions of this article is available on the website: https://www.ukbiobank.ac.uk/. Access to the UK Biobank resource could be obtained via an approved application. This study was conducted under UK Biobank application number 1094913.
