# Supplementary material for: Identifying Modifiable Factors and Their Joint Effect on Amyotrophic Lateral Sclerosis: A Prospective Cohort Study in the UK Biobank
Source: Brain Behav. 2026 Sep 23;16(9):e71771. doi: 10.1002/brb3.71771 (PMC13601743; doi:10.1002/brb3.71771)
Supplement: Supplementary file 1 [file BRB3-16-e71771-s001.docx]

**Figure S1. Modifiable factors associated with ALS in people ≥ 65 years old.**

**(A)** The x axis shows the category domains and the y axis represents statistical significance (-log10 of the *P* value). **(B)** Dots represent hazard ratios, horizontal lines indicate corresponding 95% CIs. Hazard ratios were calculated using Cox proportional hazards regression analysis after adjustments for age at baseline and sex.

**Figure S2. Modifiable factors associated with ALS in people < 65 years old.**

**(A)** The x axis shows the category domains and the y axis represents statistical significance (-log10 of the *P* value). **(B)** Dots represent hazard ratios, horizontal lines indicate corresponding 95% CIs. Hazard ratios were calculated using Cox proportional hazards regression analysis after adjustments for age at baseline and sex.

**Figure S3. Modifiable factors associated with ALS in males.**

**(A)** The x axis shows the category domains and the y axis represents statistical significance (-log10 of the *P* value). **(B)** Dots represent hazard ratios, horizontal lines indicate corresponding 95% CIs. Hazard ratios were calculated using Cox proportional hazards regression analysis after adjustments for age at baseline.

**Figure S4. Modifiable factors associated with ALS in females.**

**(A)** The x axis shows the category domains and the y axis represents statistical significance (-log10 of the *P* value). **(B)** Dots represent hazard ratios, horizontal lines indicate corresponding 95% CIs. Hazard ratios were calculated using Cox proportional hazards regression analysis after adjustments for age at baseline

**Table S1. Original data processing.**

| Variable ID | Field ID | Variables | Category | Coding | Meaning | Notes |
| --- | --- | --- | --- | --- | --- | --- |
| 1687_1 | 1687 | Comparative body size at age 10 (thinner) | Early life | 1 | Thinner | “About average” set as 0 |
| 1687_2 | 1687 | Comparative body size at age 10 (plumper) | Early life | 2 | Plumper |  |
| 1697_1 | 1697 | Comparative height size at age 10 (shorter) | Early life | 1 | Shorter | “About average” set as 0 |
| 1697_2 | 1697 | Comparative height size at age 10 (taller) | Early life | 2 | Taller |  |
| 1787 | 1787 | Maternal smoking around birth | Early life | 1 | Yes | “No” set as 0 |
| 1050_1 | 1050 | Time spent outdoors in summer (medium) | Lifestyles | 1 | Middle group | “Lower group” set as 0 |
| 1050_2 | 1050 | Time spent outdoors in summer (long) | Lifestyles | 2 | Upper group |  |
| 1060_1 | 1060 | Time spent outdoors in winter (medium) | Lifestyles | 1 | Middle group | “Lower group” set as 0 |
| 1060_2 | 1060 | Time spent outdoors in winter (long) | Lifestyles | 2 | Upper group |  |
| 1070_1 | 1070 | Time spent watching television (medium) | Lifestyles | 1 | Middle group | “Lower group” set as 0 |
| 1070_2 | 1070 | Time spent watching television (long) | Lifestyles | 2 | Upper group |  |
| 1080_1 | 1080 | Time spent using computer (short) | Lifestyles | 1 | Lower group | “Middle group” set as 0 |
| 1080_2 | 1080 | Time spent using computer (long) | Lifestyles | 2 | Upper group |  |
| 1130_1 | 1130 | Hands-free device/speakerphone use with mobile phone in last 3 month (medium) | Lifestyles | 1 | About half the time | “Never or almost never” set as 0 |
| 1130_2 | 1130 | Hands-free device/speakerphone use with mobile phone in last 3 month (long) | Lifestyles | 2 | About half the time; More than half the time; Always or almost always |  |
| 1190_1 | 1190 | Nap during day (medium) | Lifestyles | 1 | Sometimes | “Never/rarely” set as 0 |
| 1190_2 | 1190 | Nap during day (long) | Lifestyles | 2 | Usually |  |
| 1200_1 | 1200 | Sleeplessness / insomnia (sometimes) | Lifestyles | 1 | Sometimes | “Never/rarely” set as 0 |
| 1200_2 | 1200 | Sleeplessness / insomnia (usually) | Lifestyles | 2 | Usually |  |
| 1210 | 1210 | Snoring | Lifestyles | 1 | Yes | “No” set as 0 |
| 1269 | 1269 | Exposure to tobacco smoke at home | Lifestyles | 1 | Yes | “No” set as 0 |
| 1279 | 1279 | Exposure to tobacco smoke outside home | Lifestyles | 1 | Yes | “No” set as 0 |
| 1289_1 | 1289 | Cooked vegetable intake (medium) | Lifestyles | 1 | Middle group | “Lower group” set as 0 |
| 1289_2 | 1289 | Cooked vegetable intake (high) | Lifestyles | 2 | Upper group |  |
| 1299_1 | 1299 | Salad / raw vegetable intake (medium) | Lifestyles | 1 | Middle group | “Lower group” set as 0 |
| 1299_2 | 1299 | Salad / raw vegetable intake (high) | Lifestyles | 2 | Upper group |  |
| 1309_1 | 1309 | Fresh fruit intake (medium) | Lifestyles | 1 | Middle group | “Lower group” set as 0 |
| 1309_2 | 1309 | Fresh fruit intake (high) | Lifestyles | 2 | Upper group |  |
| 1319_1 | 1319 | Dried fruit intake (medium) | Lifestyles | 1 | Middle group | “Lower group” set as 0 |
| 1319_2 | 1319 | Dried fruit intake (high) | Lifestyles | 2 | Upper group |  |
| 1329_1 | 1329 | Oily fish intake (less than once a week) | Lifestyles | 1 | Less than once a week | “Never” set as 0 |
| 1329_2 | 1329 | Oily fish intake (once a week or more) | Lifestyles | 2 | Once a week; 2-4 times a week; 5-6 times a week; Once or more daily |  |
| 1339_1 | 1339 | Non-oily fish intake (less than once a week) | Lifestyles | 1 | Less than once a week | “Never” set as 0 |
| 1339_2 | 1339 | Non-oily fish intake (once a week or more) | Lifestyles | 2 | Once a week; 2-4 times a week; 5-6 times a week; Once or more daily |  |
| 1349_1 | 1349 | Processed meat intake (less than once a week) | Lifestyles | 1 | Less than once a week | “Never” set as 0 |
| 1349_2 | 1349 | Processed meat intake (once a week or more) | Lifestyles | 2 | Once a week; 2-4 times a week; 5-6 times a week; Once or more daily |  |
| 1359_1 | 1359 | Poultry intake (less than once a week) | Lifestyles | 1 | Less than once a week | “Never” set as 0 |
| 1359_2 | 1359 | Poultry intake (once a week or more) | Lifestyles | 2 | Once a week; 2-4 times a week; 5-6 times a week; Once or more daily |  |
| 1369_1 | 1369 | Beef intake (less than once a week) | Lifestyles | 1 | Less than once a week | “Never” set as 0 |
| 1369_2 | 1369 | Beef intake (once a week or more) | Lifestyles | 2 | Once a week; 2-4 times a week; 5-6 times a week; Once or more daily |  |
| 1379_1 | 1379 | Lamb/mutton intake (less than once a week) | Lifestyles | 1 | Less than once a week | “Never” set as 0 |
| 1379_2 | 1379 | Lamb/mutton intake (once a week or more) | Lifestyles | 2 | Once a week; 2-4 times a week; 5-6 times a week; Once or more daily |  |
| 1389_1 | 1389 | Pork intake (less than once a week) | Lifestyles | 1 | Less than once a week | “Never” set as 0 |
| 1389_2 | 1389 | Pork intake (once a week or more) | Lifestyles | 2 | Once a week; 2-4 times a week; 5-6 times a week; Once or more daily |  |
| 1408_1 | 1408 | Cheese intake (less than once a week) | Lifestyles | 1 | Less than once a week | “Never” set as 0 |
| 1408_2 | 1408 | Cheese intake (once a week or more) | Lifestyles | 2 | Once a week; 2-4 times a week; 5-6 times a week; Once or more daily |  |
| 1438_1 | 1438 | Bread intake (medium) | Lifestyles | 1 | Middle group | “Lower group” set as 0 |
| 1438_2 | 1438 | Bread intake (high) | Lifestyles | 2 | Upper group |  |
| 1458_1 | 1458 | Cereal intake (medium) | Lifestyles | 1 | Middle group | “Lower group” set as 0 |
| 1458_2 | 1458 | Cereal intake (high) | Lifestyles | 2 | Upper group |  |
| 1498_1 | 1498 | Coffee intake (medium) | Lifestyles | 1 | Middle group | “Lower group” set as 0 |
| 1498_2 | 1498 | Coffee intake (high) | Lifestyles | 2 | Upper group |  |
| 1528_1 | 1528 | Water intake (medium) | Lifestyles | 1 | Middle group | “Lower group” set as 0 |
| 1528_2 | 1528 | Water intake (high) | Lifestyles | 2 | Upper group |  |
| 1558_1 | 1558 | Alcohol intake frequency (less than once a week) | Lifestyles | 1 | Special occasions only; One to three times a month | “Never” set as 0 |
| 1558_2 | 1558 | Alcohol intake frequency (once a week or more) | Lifestyles | 2 | Once or twice a week; Three or four times a week; Daily or almost daily |  |
| 20160 | 20160 | Ever smoked | Lifestyles | 1 | Yes | “No” set as 0 |
| 2139_1 | 2139 | Age first had sexual intercourse (medium) | Lifestyles | 1 | Middle group | “Lower group” set as 0 |
| 2139_2 | 2139 | Age first had sexual intercourse (late) | Lifestyles | 2 | Upper group |  |
| 2149_1 | 2149 | Lifetime number of sexual partners (medium) | Lifestyles | 1 | Middle group | “Lower group” set as 0 |
| 2149_2 | 2149 | Lifetime number of sexual partners (more) | Lifestyles | 2 | Upper group |  |
| 2159 | 2159 | Ever had same-sex intercourse | Lifestyles | 1 | Yes | “No” set as 0 |
| 22037_1 | 22037 | MET minutes per week for walking (medium) | Lifestyles | 1 | Middle group | “Lower group” set as 0 |
| 22037_2 | 22037 | MET minutes per week for walking (long) | Lifestyles | 2 | Upper group |  |
| 22038_1 | 22038 | MET minutes per week for moderate activity (medium) | Lifestyles | 1 | Middle group | “Lower group” set as 0 |
| 22038_2 | 22038 | MET minutes per week for moderate activity (long) | Lifestyles | 2 | Upper group |  |
| 22039_1 | 22039 | MET minutes per week for vigorous activity (medium) | Lifestyles | 1 | Middle group | “Lower group” set as 0 |
| 22039_2 | 22039 | MET minutes per week for vigorous activity (long) | Lifestyles | 2 | Upper group |  |
| 6144_1 | 6144_1 | Intake of eggs or food containing eggs | Lifestyles | 1 | Yes | “No” set as 0 |
| 6144_2 | 6144_2 | Intake of dairy product | Lifestyles | 1 | Yes | “No” set as 0 |
| 6144_3 | 6144_3 | Intake of wheat product | Lifestyles | 1 | Yes | “No” set as 0 |
| 6144_4 | 6144_4 | Intake of sugar or foods/drinks containing sugar | Lifestyles | 1 | Yes | “No” set as 0 |
| 6155_1 | 6155_1 | Intake of vitamin A supplements | Lifestyles | 1 | Yes | “No” set as 0 |
| 6155_2 | 6155_2 | Intake of vitamin B supplements | Lifestyles | 1 | Yes | “No” set as 0 |
| 6155_3 | 6155_3 | Intake of vitamin C supplements | Lifestyles | 1 | Yes | “No” set as 0 |
| 6155_4 | 6155_4 | Intake of vitamin D supplements | Lifestyles | 1 | Yes | “No” set as 0 |
| 6155_5 | 6155_5 | Intake of vitamin E supplements | Lifestyles | 1 | Yes | “No” set as 0 |
| 6155_6 | 6155_6 | Intake of folic acid or Folate (Vit B9) supplements | Lifestyles | 1 | Yes | “No” set as 0 |
| 6155_7 | 6155_7 | Intake of multivitamins +/- minerals supplements | Lifestyles | 1 | Yes | “No” set as 0 |
| 6160_1 | 6160_1 | Join in sports club or gym | Lifestyles | 1 | Yes | “No” set as 0 |
| 6160_2 | 6160_2 | Join in pub or social club | Lifestyles | 1 | Yes | “No” set as 0 |
| 6160_3 | 6160_3 | Join in religious group | Lifestyles | 1 | Yes | “No” set as 0 |
| 6160_4 | 6160_4 | Join in adult education class | Lifestyles | 1 | Yes | “No” set as 0 |
| 6160_5 | 6160_5 | Join in other group activity | Lifestyles | 1 | Yes | “No” set as 0 |
| 6179_1 | 6179_1 | Intake of fish oil (including cod liver oil) supplements | Lifestyles | 1 | Yes | “No” set as 0 |
| 6179_2 | 6179_2 | Intake of glucosamine supplements | Lifestyles | 1 | Yes | “No” set as 0 |
| 6179_3 | 6179_3 | Intake of calcium supplements | Lifestyles | 1 | Yes | “No” set as 0 |
| 6179_4 | 6179_4 | Intake of zinc supplements | Lifestyles | 1 | Yes | “No” set as 0 |
| 6179_5 | 6179_5 | Intake of iron supplements | Lifestyles | 1 | Yes | “No” set as 0 |
| 6179_6 | 6179_6 | Intake of selenium supplements | Lifestyles | 1 | Yes | “No” set as 0 |
| 24004_1 | 24004 | Nitrogen oxides air pollution (medium) | Local environment | 1 | Middle group | “Lower group” set as 0 |
| 24004_2 | 24004 | Nitrogen oxides air pollution (high) | Local environment | 2 | Upper group |  |
| 24005_1 | 24005 | Particulate matter air pollution pm10 (medium) | Local environment | 1 | Middle group | “Lower group” set as 0 |
| 24005_2 | 24005 | Particulate matter air pollution pm10 (high) | Local environment | 2 | Upper group |  |
| 24006_1 | 24006 | Particulate matter air pollution pm2.5 (medium) | Local environment | 1 | Middle group | “Lower group” set as 0 |
| 24006_2 | 24006 | Particulate matter air pollution pm2.5 (high) | Local environment | 2 | Upper group |  |
| 24008_1 | 24008 | Particulate matter air pollution 2.5-10um (medium) | Local environment | 1 | Middle group | “Lower group” set as 0 |
| 24008_2 | 24008 | Particulate matter air pollution 2.5-10um (high) | Local environment | 2 | Upper group |  |
| 24020_1 | 24020 | Average daytime sound level of noise pollution (medium) | Local environment | 1 | Middle group | “Lower group” set as 0 |
| 24020_2 | 24020 | Average daytime sound level of noise pollution (high) | Local environment | 2 | Upper group |  |
| 24021_1 | 24021 | Average evening sound level of noise pollution (medium) | Local environment | 1 | Middle group | “Lower group” set as 0 |
| 24021_2 | 24021 | Average evening sound level of noise pollution (high) | Local environment | 2 | Upper group |  |
| 24022_1 | 24022 | Average night-time sound level of noise pollution (medium) | Local environment | 1 | Middle group | “Lower group” set as 0 |
| 24022_2 | 24022 | Average night-time sound level of noise pollution (high) | Local environment | 2 | Upper group |  |
| 24024_1 | 24024 | Average 24-hour sound level of noise pollution (medium) | Local environment | 1 | Middle group | “Lower group” set as 0 |
| 24024_2 | 24024 | Average 24-hour sound level of noise pollution (high) | Local environment | 2 | Upper group |  |
| 24500_1 | 24500 | Greenspace percentage, buffer 1000m (medium) | Local environment | 1 | Middle group | “Lower group” set as 0 |
| 24500_2 | 24500 | Greenspace percentage, buffer 1000m (high) | Local environment | 2 | Upper group |  |
| 24501_1 | 24501 | Domestic garden percentage, buffer 1000m (medium) | Local environment | 1 | Middle group | “Lower group” set as 0 |
| 24501_2 | 24501 | Domestic garden percentage, buffer 1000m (high) | Local environment | 2 | Upper group |  |
| 24502_1 | 24502 | Water percentage, buffer 1000m (medium) | Local environment | 1 | Middle group | “Lower group” set as 0 |
| 24502_2 | 24502 | Water percentage, buffer 1000m (high) | Local environment | 2 | Upper group |  |
| 24506_1 | 24506 | Natural environment percentage, buffer 1000m (medium) | Local environment | 1 | Middle group | “Lower group” set as 0 |
| 24506_2 | 24506 | Natural environment percentage, buffer 1000m (high) | Local environment | 2 | Upper group |  |
| 130176 | 130176 | Varicella | Medical history | 1 | Yes | “No” set as 0 |
| 130178 | 130178 | Zoster | Medical history | 1 | Yes | “No” set as 0 |
| 130184 | 130184 | Measles | Medical history | 1 | Yes | “No” set as 0 |
| 130186 | 130186 | Rubella | Medical history | 1 | Yes | “No” set as 0 |
| 130188 | 130188 | Viral warts | Medical history | 1 | Yes | “No” set as 0 |
| 130216 | 130216 | Mumps | Medical history | 1 | Yes | “No” set as 0 |
| 130226 | 130226 | Dermatophytosis | Medical history | 1 | Yes | “No” set as 0 |
| 130230 | 130230 | Candidiasis | Medical history | 1 | Yes | “No” set as 0 |
| 130622 | 130622 | Iron deficiency anaemia | Medical history | 1 | Yes | “No” set as 0 |
| 130814 | 130814 | Disorders of lipoprotein metabolism and other lipidaemias | Medical history | 1 | Yes | “No” set as 0 |
| 130820 | 130820 | Disorders of mineral metabolism | Medical history | 1 | Yes | “No” set as 0 |
| 130854 | 130854 | Mental and behavioural disorders due to use of alcohol | Medical history | 1 | Yes | “No” set as 0 |
| 130868 | 130868 | Mental and behavioural disorders due to use of tobacco | Medical history | 1 | Yes | “No” set as 0 |
| 130894 | 130894 | Depressive episode | Medical history | 1 | Yes | “No” set as 0 |
| 130910 | 130910 | Reaction to severe stress, and adjustment disorders | Medical history | 1 | Yes | “No” set as 0 |
| 131052 | 131052 | Migraine | Medical history | 1 | Yes | “No” set as 0 |
| 131056 | 131056 | Transient cerebral ischaemic attacks and related syndromes | Medical history | 1 | Yes | “No” set as 0 |
| 131060 | 131060 | Sleep disorders | Medical history | 1 | Yes | “No” set as 0 |
| 131074 | 131074 | Mononeuropathies of upper limb | Medical history | 1 | Yes | “No” set as 0 |
| 131128 | 131128 | Hordeolum and chalazion | Medical history | 1 | Yes | “No” set as 0 |
| 131136 | 131136 | Disorders of lachrymal system | Medical history | 1 | Yes | “No” set as 0 |
| 131142 | 131142 | Conjunctivitis | Medical history | 1 | Yes | “No” set as 0 |
| 131164 | 131164 | Senile cataract | Medical history | 1 | Yes | “No” set as 0 |
| 131186 | 131186 | Glaucoma | Medical history | 1 | Yes | “No” set as 0 |
| 131190 | 131190 | Disorders of vitreous body | Medical history | 1 | Yes | “No” set as 0 |
| 131208 | 131208 | Disorders of refraction and accommodation | Medical history | 1 | Yes | “No” set as 0 |
| 131210 | 131210 | Visual disturbances | Medical history | 1 | Yes | “No” set as 0 |
| 131222 | 131222 | Otitis externa | Medical history | 1 | Yes | “No” set as 0 |
| 131252 | 131252 | Disorders of vestibular function | Medical history | 1 | Yes | “No” set as 0 |
| 131262 | 131262 | Otalgia and effusion of ear | Medical history | 1 | Yes | “No” set as 0 |
| 131286 | 131286 | Essential (primary) hypertension | Medical history | 1 | Yes | “No” set as 0 |
| 131296 | 131296 | Angina pectoris | Medical history | 1 | Yes | “No” set as 0 |
| 131298 | 131298 | Acute myocardial infarction | Medical history | 1 | Yes | “No” set as 0 |
| 131306 | 131306 | Chronic ischaemic heart disease | Medical history | 1 | Yes | “No” set as 0 |
| 131308 | 131308 | Pulmonary embolism | Medical history | 1 | Yes | “No” set as 0 |
| 131350 | 131350 | Atrial fibrillation and flutter | Medical history | 1 | Yes | “No” set as 0 |
| 131354 | 131354 | Heart failure | Medical history | 1 | Yes | “No” set as 0 |
| 131366 | 131366 | Cerebral infarction | Medical history | 1 | Yes | “No” set as 0 |
| 131396 | 131396 | Phlebitis and thrombophlebitis | Medical history | 1 | Yes | “No” set as 0 |
| 131402 | 131402 | Varicose veins of lower extremities | Medical history | 1 | Yes | “No” set as 0 |
| 131404 | 131404 | Haemorrhoids | Medical history | 1 | Yes | “No” set as 0 |
| 131416 | 131416 | Hypotension | Medical history | 1 | Yes | “No” set as 0 |
| 131426 | 131426 | Acute sinusitis | Medical history | 1 | Yes | “No” set as 0 |
| 131428 | 131428 | Acute pharyngitis | Medical history | 1 | Yes | “No” set as 0 |
| 131430 | 131430 | Acute tonsillitis | Medical history | 1 | Yes | “No” set as 0 |
| 131442 | 131442 | Influenza | Medical history | 1 | Yes | “No” set as 0 |
| 131456 | 131456 | Pneumonia | Medical history | 1 | Yes | “No” set as 0 |
| 131464 | 131464 | Vasomotor and allergic rhinitis | Medical history | 1 | Yes | “No” set as 0 |
| 131468 | 131468 | Chronic sinusitis | Medical history | 1 | Yes | “No” set as 0 |
| 131484 | 131484 | Bronchitis | Medical history | 1 | Yes | “No” set as 0 |
| 131494 | 131494 | Asthma | Medical history | 1 | Yes | “No” set as 0 |
| 131582 | 131582 | Oesophagitis | Medical history | 1 | Yes | “No” set as 0 |
| 131584 | 131584 | Gastro-oesophageal reflux disease | Medical history | 1 | Yes | “No” set as 0 |
| 131590 | 131590 | Gastric ulcer | Medical history | 1 | Yes | “No” set as 0 |
| 131592 | 131592 | Duodenal ulcer | Medical history | 1 | Yes | “No” set as 0 |
| 131598 | 131598 | Gastritis and duodenitis | Medical history | 1 | Yes | “No” set as 0 |
| 131600 | 131600 | Dyspepsia | Medical history | 1 | Yes | “No” set as 0 |
| 131612 | 131612 | Inguinal hernia | Medical history | 1 | Yes | “No” set as 0 |
| 131620 | 131620 | Diaphragmatic hernia | Medical history | 1 | Yes | “No” set as 0 |
| 131634 | 131634 | Paralytic ileus and intestinal obstruction without hernia | Medical history | 1 | Yes | “No” set as 0 |
| 131636 | 131636 | Diverticular disease of intestine | Medical history | 1 | Yes | “No” set as 0 |
| 131638 | 131638 | Irritable bowel syndrome | Medical history | 1 | Yes | “No” set as 0 |
| 131674 | 131674 | Cholelithiasis | Medical history | 1 | Yes | “No” set as 0 |
| 131700 | 131700 | Cutaneous abscess, furuncle and carbuncle | Medical history | 1 | Yes | “No” set as 0 |
| 131702 | 131702 | Cellulitis | Medical history | 1 | Yes | “No” set as 0 |
| 131720 | 131720 | Atopic dermatitis | Medical history | 1 | Yes | “No” set as 0 |
| 131722 | 131722 | Seborrhoeic dermatitis | Medical history | 1 | Yes | “No” set as 0 |
| 131738 | 131738 | Pruritus | Medical history | 1 | Yes | “No” set as 0 |
| 131742 | 131742 | Psoriasis | Medical history | 1 | Yes | “No” set as 0 |
| 131754 | 131754 | Urticaria | Medical history | 1 | Yes | “No” set as 0 |
| 131794 | 131794 | Follicular cysts of skin and subcutaneous tissue | Medical history | 1 | Yes | “No” set as 0 |
| 131806 | 131806 | Seborrhoeic keratosis | Medical history | 1 | Yes | “No” set as 0 |
| 131858 | 131858 | Gout | Medical history | 1 | Yes | “No” set as 0 |
| 131868 | 131868 | Polyarthrosis | Medical history | 1 | Yes | “No” set as 0 |
| 131870 | 131870 | Coxarthrosis | Medical history | 1 | Yes | “No” set as 0 |
| 131872 | 131872 | Gonarthrosis | Medical history | 1 | Yes | “No” set as 0 |
| 131878 | 131878 | Acquired deformities of fingers and toes | Medical history | 1 | Yes | “No” set as 0 |
| 131916 | 131916 | Spondylosis | Medical history | 1 | Yes | “No” set as 0 |
| 131928 | 131928 | Dorsalgia | Medical history | 1 | Yes | “No” set as 0 |
| 131938 | 131938 | Synovitis and tenosynovitis | Medical history | 1 | Yes | “No” set as 0 |
| 131946 | 131946 | Soft tissue disorders related to use, overuse and pressure | Medical history | 1 | Yes | “No” set as 0 |
| 131950 | 131950 | Fibroblastic disorders | Medical history | 1 | Yes | “No” set as 0 |
| 131964 | 131964 | Osteoporosis without pathological fracture | Medical history | 1 | Yes | “No” set as 0 |
| 132032 | 132032 | Chronic renal failure | Medical history | 1 | Yes | “No” set as 0 |
| 132036 | 132036 | Calculus of kidney and ureter | Medical history | 1 | Yes | “No” set as 0 |
| 132054 | 132054 | Cystitis | Medical history | 1 | Yes | “No” set as 0 |
| 23099_1 | 23099 | Body fat percentage (medium) | Physical measures | 1 | Middle group | “Lower group” set as 0 |
| 23099_2 | 23099 | Body fat percentage (high) | Physical measures | 2 | Upper group |  |
| 23105_1 | 23105 | Basal metabolic rate (medium) | Physical measures | 1 | Middle group | “Lower group” set as 0 |
| 23105_2 | 23105 | Basal metabolic rate (high) | Physical measures | 2 | Upper group |  |
| 23111_23115_1 | 23111/23115 | Leg fat percentage (medium) | Physical measures | 1 | Middle group | “Lower group” set as 0 |
| 23111_23115_2 | 23111/23115 | Leg fat percentage (high) | Physical measures | 2 | Upper group | Mean value of left and right |
| 23119_23123_1 | 23119/23123 | Arm fat percentage (medium) | Physical measures | 1 | Middle group | “Lower group” set as 0 |
| 23119_23123_2 | 23119/23123 | Arm fat percentage (high) | Physical measures | 2 | Upper group | Mean value of left and right |
| 23127_1 | 23127 | Trunk fat percentage (medium) | Physical measures | 1 | Middle group | “Lower group” set as 0 |
| 23127_2 | 23127 | Trunk fat percentage (high) | Physical measures | 2 | Upper group |  |
| 3062_1 | 3062 | Forced vital capacity (medium) | Physical measures | 1 | Middle group | “Lower group” set as 0 |
| 3062_2 | 3062 | Forced vital capacity (high) | Physical measures | 2 | Upper group |  |
| 3063_1 | 3063 | Forced expiratory volume in 1-second (medium) | Physical measures | 1 | Middle group | “Lower group” set as 0 |
| 3063_2 | 3063 | Forced expiratory volume in 1-second (high) | Physical measures | 2 | Upper group |  |
| 3064_1 | 3064 | Peak expiratory flow (medium) | Physical measures | 1 | Middle group | “Lower group” set as 0 |
| 3064_2 | 3064 | Peak expiratory flow (high) | Physical measures | 2 | Upper group |  |
| 4079_1 | 4079 | Diastolic blood pressure (medium) | Physical measures | 1 | Middle group | “Lower group” set as 0 |
| 4079_2 | 4079 | Diastolic blood pressure (high) | Physical measures | 2 | Upper group |  |
| 4080_1 | 4080 | Systolic blood pressure (medium) | Physical measures | 1 | Middle group | “Lower group” set as 0 |
| 4080_2 | 4080 | Systolic blood pressure (high) | Physical measures | 2 | Upper group |  |
| 46_47_1 | 46/47 | Hand grip strength (medium) | Physical measures | 1 | Middle group | “Lower group” set as 0 |
| 46_47_2 | 46/47 | Hand grip strength (high) | Physical measures | 2 | Upper group | Mean value of left and right |
| 1920 | 1920 | Mood swings | Psychosocial factors | 1 | Yes | “No” set as 0 |
| 1930 | 1930 | Miserableness | Psychosocial factors | 1 | Yes | “No” set as 0 |
| 1940 | 1940 | Irritability | Psychosocial factors | 1 | Yes | “No” set as 0 |
| 1950 | 1950 | Sensitivity / hurt feelings | Psychosocial factors | 1 | Yes | “No” set as 0 |
| 1960 | 1960 | Fed-up feelings | Psychosocial factors | 1 | Yes | “No” set as 0 |
| 1970 | 1970 | Nervous feelings | Psychosocial factors | 1 | Yes | “No” set as 0 |
| 1980 | 1980 | Worrier / anxious feelings | Psychosocial factors | 1 | Yes | “No” set as 0 |
| 1990 | 1990 | Tense / 'highly strung' | Psychosocial factors | 1 | Yes | “No” set as 0 |
| 2000 | 2000 | Worry too long after embarrassment | Psychosocial factors | 1 | Yes | “No” set as 0 |
| 2010 | 2010 | Suffer from 'nerves' | Psychosocial factors | 1 | Yes | “No” set as 0 |
| 2020 | 2020 | Loneliness, isolation | Psychosocial factors | 1 | Yes | “No” set as 0 |
| 2030 | 2030 | Guilty feelings | Psychosocial factors | 1 | Yes | “No” set as 0 |
| 22189_1 | 22189 | Townsend deprivation index at recruitment (medium) | SES | 1 | Middle group | “Lower group” set as 0 |
| 22189_2 | 22189 | Townsend deprivation index at recruitment (high) | SES | 2 | Upper group |  |
| 6138 | 6138 | Lower education | SES | 1 | A levels/AS levels or equivalent; O levels/GCSEs or equivalent; CSEs or equivalent; None of the above | "College or University degree; Other professional qualifications" set as 0 |
| 6142 | 6142 | Unemployed | SES | 1 | Looking after home and/or family; Unable to work because of sickness or disability; Unemployed | "In paid employment or self-employed; Retired; Doing unpaid or voluntary work; Full or part-time student" set as 0 |
| 738_1 | 738 | Average total household income before tax (18,000 to 30,999) | SES | 1 | 18,000 to 30,999 | “Less than 18,000” set as 0 |
| 738_2 | 738 | Average total household income before tax (31,000 to 51,999) | SES | 2 | 31,000 to 51,999 |  |
| 738_3 | 738 | Average total household income before tax (Greater than 52,000) | SES | 3 | 52,000 to 100,000; Greater than 100,000 |  |

**Table S2. Lower tertile and upper tertile of continuous variables.**

| Variable ID | Lower tertile | Upper tertile |
| --- | --- | --- |
| 24004 | 36.63 | 46.92 |
| 24005 | 15.59 | 16.57 |
| 24006 | 9.45 | 10.23 |
| 24008 | 5.93 | 6.41 |
| 24020 | 53.37 | 55.41 |
| 24021 | 49.63 | 51.67 |
| 24022 | 44.55 | 46.59 |
| 24024 | 54.01 | 56.06 |
| 24500 | 32.828 | 53.902 |
| 24501 | 19.751 | 29.048 |
| 24502 | 0.327 | 0.999 |
| 24506 | 24.94 | 52.09 |
| 1050 | 2 | 4 |
| 1060 | 1 | 2 |
| 1070 | 2 | 3 |
| 1080 | 0.5 | 1 |
| 1289 | 2 | 3 |
| 1299 | 1 | 2 |
| 1309 | 1 | 3 |
| 1319 | 0 | 1 |
| 1438 | 8 | 14 |
| 1458 | 4 | 6 |
| 1498 | 1 | 2 |
| 1528 | 1 | 3 |
| 2139 | 17.4 | 20 |
| 2149 | 2 | 6 |
| 22037 | 425.7 | 990 |
| 22038 | 240 | 840 |
| 22039 | 80 | 656 |
| 23119_23123 | 23.45 | 33.3 |
| 46_47 | 25 | 35.5 |
| 23111_23115 | 24.5 | 38.3 |
| 3062 | 3.18 | 4.03 |
| 3063 | 2.42 | 3.07 |
| 3064 | 322 | 424.67 |
| 23099 | 26.8 | 34.7 |
| 23105 | 5703 | 7201 |
| 23127 | 27.4 | 34 |
| 4079 | 77.5 | 86 |
| 4080 | 128 | 143 |
| 22189 | -3.32 | -1.07 |

**Table S3. Pathogenic genes and their rsIDs of ALS.**

| Gene | rsID |
| --- | --- |
| C9orf72 | rs143561967 |
| FUS | rs2544251972, rs387906628, rs267606832, rs387907274, rs1596908744, rs186547381, rs2144138894, rs1555509569, rs2079347087, rs2544279075, rs387906627, rs2144140268, rs1161032867, rs886041577, rs1596912983, rs2079350332, rs886041776, rs267606831, rs2544279594, rs1555509609, rs2079351113, rs2144142389, rs121909667, rs121909669, rs1567479067, rs121909668, rs121909671, rs1555509693, rs267606833, rs886041389, rs1555509699, rs886041390, rs1228194239 |
| HNRNPA1 | rs397518453, rs397518452, rs397518454, rs483353028, rs483353031, rs2540585995 |
| SOD1 | rs1568807297, rs121912444, rs121912442, rs1312702973, rs121912448, rs121912453, rs1200906022, rs1555836169, rs121912450, rs121912431, rs121912433, rs121912434, rs121912435, rs121912457, rs121912443, rs1568809172, rs1601157750, rs121912458, rs121912452, rs1315541036, rs121912436, rs11556620, rs1280042397, rs80265967, rs121912437, rs121912438, rs121912459, rs76731700, rs121912439, rs1568810715, rs121912445, rs1378590183, rs121912440, rs1568810771, rs1601158483, rs74315452, rs121912441, rs1568810789, rs1301635320, rs1555836889, rs369600566, rs1197141604, rs1568811366, rs121912454, rs1568811426, rs121912451, rs1804449, rs1568811489, rs121912446, rs1482760341, rs1555836950, rs1424014997, rs121912449 |
| TARDBP | rs80356717, rs267607102, rs80356719, rs121908395, rs80356721, rs80356723, rs4884357, rs80356726, rs80356727, rs80356730, rs80356731, rs80356732, rs80356733, rs1131690782, rs80356734, rs367543041, rs80356740, rs80356741, rs80356742, rs387906334 |
| UBQLN2 | rs1243726473, rs387906710, rs387906709, rs387906711 |
| VAPB | rs281875284, rs74315431 |
| KIF5A | rs2140150119, rs2540493214, rs2140159183, rs748551786, rs2540494891, rs2540496937, rs1488871976, rs1057524193, rs1057519195, rs1555177629, rs387907287, rs690016545, rs387907289, rs2140162541, rs2540499310, rs539166420, rs387907285, rs2140162576, rs2140162581, rs121434441, rs121434443, rs1555177824, rs121434442, rs387907288, rs2140162883, rs1416085161, rs1012819766, rs121434444, rs2540501760, rs2540503039, rs1882272161, rs185438769, rs1555178348, rs2140167500, rs2540508034, rs2540509221, rs1235560158, rs554894381, rs387907286, rs1555178616, rs1882578572, rs1594925773, rs1882598771, rs1057519078, rs1565705251, rs1057517673, rs1402429085, rs1555179091 |
| ANXA11 | rs754594235, rs1247392012, rs2492172951, rs368751524, rs142083484 |
| CHCHD10 | rs730880031, rs587777574, rs730880033, rs2145927350, rs730880030, rs730880032 |
| NEK1 | rs1157065841, rs2477550717, rs1554020563, rs1406951209, rs1732670701, rs2111335168, rs199947197, rs763310804, rs2111354717, rs2478424918, rs750159428, rs746591648, rs2546393018, rs773156346, rs2546546013, rs1131690774, rs786205645, rs1200795502, rs371575563, rs483352907, rs368940355, rs1758763118, rs2546763854, rs985064686, rs2546789977, rs765037444, rs1482234746, rs1762964297, rs1432921892, rs794727032, rs483352906, rs1554075506, rs202115635, rs2546929612, rs431905508, rs387906890, rs1049502301, rs794727285 |
| PFN1 | rs387907266, rs140547520, rs387907265, rs387907264 |
| TBK1 | rs1555201919, rs1279619378, rs1592350883, rs1592350887, rs2539482429, rs2539489004, rs1565814492, rs1565814529, rs2539493394, rs1592362719, rs2539503647, rs2539510971, rs2539511024, rs2539511036, rs1284582102, rs369620088, rs755950225, rs1341055534, rs1328949478, rs758357594, rs2539519111, rs2040816609, rs2539520026, rs769588220, rs142030898, rs2136079548, rs767898276, rs876657404, rs2539523784, rs1178717934, rs1476919390, rs1555204731, rs1280163869, rs2539534664, rs2136087722, rs2040932099, rs2136088227, rs2539537276, rs2539537514, rs1402092579, rs2136088420, rs2539537579, rs2136088934, rs748112833, rs876657406 |
| VCP | rs2490348146, rs387906790, rs767379602, rs1828721782, rs121909331, rs121909334, rs1554668805, rs121909335, rs387906789, rs1554668813, rs1554668814, rs1554668817, rs121909329, rs121909330, rs864309502, rs121909332, rs1554669087, rs863225291, rs397507560, rs1420316004, rs1829035889 |
| OPTN | rs753966040, rs934287314, rs28939688, rs1564354968, rs1358641287, rs1359892832, rs11258194, rs774258585, rs140599944, rs2539041527, rs1283443715, rs1401721711, rs771268520, rs1564360451, rs1328890259, rs2539056279, rs1371904281, rs757559365, rs750571210, rs786205611, rs142812715, rs778911925, rs746420444, rs1833438306, rs1564365123, rs267606928, rs1657737496, rs1833451208, rs774772178, rs1206478143, rs1206423781, rs1588449569, rs2539070896, rs1200521614, rs267606929, rs757107215, rs780777015, rs75654767 |

**Table S4. Univariate Cox analysis of covariates.**

| Variable | HR | CI5 | CI95 | *P* value | *P* value of  Schoenfeld residuals |
| --- | --- | --- | --- | --- | --- |
| Age | 1.1146 | 1.1017 | 1.1277 | 1.23e-74 | 0.04 |
| Sex | 1.5542 | 1.3451 | 1.7958 | 2.20e-9 | 0.05 |
| Gene | 1.1113 | 0.8271 | 1.4932 | 0.48 | 0.66 |

HR, hazard ratio; CI, confidence interval.

**Table S5. Associations between 184 risk factors and ALS by EWAS.**

| Variable ID | Variables | HR | CI5 | CI95 | *P* value | *P* value of  Schoenfeld residuals | Category |
| --- | --- | --- | --- | --- | --- | --- | --- |
| 1687_1 | Comparative body size at age 10 (thinner) | 1.0548 | 0.8987 | 1.238 | 0.513702747 | 0.5554 | Early life |
| 1687_2 | Comparative body size at age 10 (plumper) | 1.1546 | 0.9385 | 1.4205 | 0.174058998 | 0.5554 | Early life |
| 1697_1 | Comparative height size at age 10 (shorter) | 0.9337 | 0.7697 | 1.1328 | 0.48682421 | 0.7197 | Early life |
| 1697_2 | Comparative height size at age 10 (taller) | 1.0226 | 0.8633 | 1.2113 | 0.796293973 | 0.7197 | Early life |
| 1787 | Maternal smoking around birth | 1.0212 | 0.8674 | 1.2022 | 0.801445307 | 0.7903 | Early life |
| 1050_1 | Time spent outdoors in summer (medium) | 1.0459 | 0.8707 | 1.2563 | 0.631672277 | 0.9443 | Lifestyles |
| 1050_2 | Time spent outdoors in summer (long) | 1.2144 | 1.0115 | 1.4581 | 0.037331898 | 0.9443 | Lifestyles |
| 1060_1 | Time spent outdoors in winter (medium) | 1.1021 | 0.9236 | 1.3152 | 0.280879352 | 0.7049 | Lifestyles |
| 1060_2 | Time spent outdoors in winter (long) | 1.395 | 1.1696 | 1.6638 | 0.000213584 | 0.7049 | Lifestyles |
| 1070_1 | Time spent watching television (medium) | 1.1426 | 0.9508 | 1.3731 | 0.155173868 | 0.4062 | Lifestyles |
| 1070_2 | Time spent watching television (long) | 1.4421 | 1.219 | 1.7059 | 0.000019485 | 0.4062 | Lifestyles |
| 1080_1 | Time spent using computer (short) | 1.3731 | 1.1543 | 1.6335 | 0.000343532 | 0.394 | Lifestyles |
| 1080_2 | Time spent using computer (long) | 1.0855 | 0.8933 | 1.3189 | 0.409285179 | 0.394 | Lifestyles |
| 1130_1 | Hands-free device/speakerphone use with mobile phone in last 3 month (medium) | 1.0075 | 0.7618 | 1.3325 | 0.958117494 | 0.1163 | Lifestyles |
| 1130_2 | Hands-free device/speakerphone use with mobile phone in last 3 month (long) | 1.1322 | 0.8426 | 1.5214 | 0.410161067 | 0.1163 | Lifestyles |
| 1190_1 | Nap during day (medium) | 1.0078 | 0.8654 | 1.1737 | 0.920175435 | 0.3432 | Lifestyles |
| 1190_2 | Nap during day (long) | 1.14 | 0.8459 | 1.5363 | 0.389544134 | 0.3432 | Lifestyles |
| 1200_1 | Sleeplessness / insomnia (sometimes) | 1.0968 | 0.9111 | 1.3205 | 0.32887245 | 0.3081 | Lifestyles |
| 1200_2 | Sleeplessness / insomnia (usually) | 1.1766 | 0.9601 | 1.4419 | 0.116951461 | 0.3081 | Lifestyles |
| 1210 | Snoring | 0.8541 | 0.732 | 0.9965 | 0.045037784 | 0.2499 | Lifestyles |
| 1269 | Exposure to tobacco smoke at home | 1.3696 | 0.9958 | 1.8836 | 0.053089336 | 0.2302 | Lifestyles |
| 1279 | Exposure to tobacco smoke outside home | 1.0909 | 0.9003 | 1.3217 | 0.374531942 | 0.5954 | Lifestyles |
| 1289_1 | Cooked vegetable intake (medium) | 1.0647 | 0.8995 | 1.2603 | 0.465963867 | 0.0029 | Lifestyles |
| 1289_2 | Cooked vegetable intake (high) | 1.0855 | 0.9039 | 1.3035 | 0.379732231 | 0.0029 | Lifestyles |
| 1299_1 | Salad / raw vegetable intake (medium) | 0.9652 | 0.8062 | 1.1555 | 0.699446608 | 0.7503 | Lifestyles |
| 1299_2 | Salad / raw vegetable intake (high) | 0.9293 | 0.7832 | 1.1027 | 0.401052868 | 0.7503 | Lifestyles |
| 1309_1 | Fresh fruit intake (medium) | 0.8947 | 0.7612 | 1.0516 | 0.177178579 | 0.2594 | Lifestyles |
| 1309_2 | Fresh fruit intake (high) | 0.9441 | 0.7608 | 1.1715 | 0.601298642 | 0.2594 | Lifestyles |
| 1319_1 | Dried fruit intake (medium) | 0.8385 | 0.6983 | 1.0069 | 0.059285289 | 0.6802 | Lifestyles |
| 1319_2 | Dried fruit intake (high) | 0.8804 | 0.7278 | 1.0651 | 0.189884115 | 0.6802 | Lifestyles |
| 1329_1 | Oily fish intake (less than once a week) | 0.7463 | 0.5755 | 0.9678 | 0.027334041 | 0.6653 | Lifestyles |
| 1329_2 | Oily fish intake (once a week or more) | 0.6926 | 0.5408 | 0.8871 | 0.003624379 | 0.6653 | Lifestyles |
| 1339_1 | Non-oily fish intake (less than once a week) | 0.6895 | 0.4738 | 1.0034 | 0.052132406 | 0.3725 | Lifestyles |
| 1339_2 | Non-oily fish intake (once a week or more) | 0.8064 | 0.5638 | 1.1533 | 0.23854294 | 0.3725 | Lifestyles |
| 1349_1 | Processed meat intake (less than once a week) | 0.7461 | 0.5746 | 0.9688 | 0.027979674 | 0.0744 | Lifestyles |
| 1349_2 | Processed meat intake (once a week or more) | 0.8075 | 0.6302 | 1.0348 | 0.091083465 | 0.0744 | Lifestyles |
| 1359_1 | Poultry intake (less than once a week) | 0.9525 | 0.6539 | 1.3875 | 0.799851221 | 0.2749 | Lifestyles |
| 1359_2 | Poultry intake (once a week or more) | 0.8328 | 0.5995 | 1.1569 | 0.27527469 | 0.2749 | Lifestyles |
| 1369_1 | Beef intake (less than once a week) | 0.7104 | 0.5609 | 0.8998 | 0.004574413 | 0.2902 | Lifestyles |
| 1369_2 | Beef intake (once a week or more) | 0.7633 | 0.602 | 0.9678 | 0.025707086 | 0.2902 | Lifestyles |
| 1379_1 | Lamb/mutton intake (less than once a week) | 0.7872 | 0.6433 | 0.9632 | 0.020144186 | 0.7373 | Lifestyles |
| 1379_2 | Lamb/mutton intake (once a week or more) | 0.881 | 0.7046 | 1.1015 | 0.266120152 | 0.7373 | Lifestyles |
| 1389_1 | Pork intake (less than once a week) | 0.7763 | 0.6332 | 0.9517 | 0.014852625 | 0.3378 | Lifestyles |
| 1389_2 | Pork intake (once a week or more) | 0.9226 | 0.7366 | 1.1555 | 0.483072582 | 0.3378 | Lifestyles |
| 1408_1 | Cheese intake (less than once a week) | 0.9737 | 0.5939 | 1.5962 | 0.915696695 | 0.6431 | Lifestyles |
| 1408_2 | Cheese intake (once a week or more) | 0.8584 | 0.5371 | 1.3718 | 0.52314461 | 0.6431 | Lifestyles |
| 1438_1 | Bread intake (medium) | 0.9493 | 0.7967 | 1.1312 | 0.560959216 | 0.0024 | Lifestyles |
| 1438_2 | Bread intake (high) | 1.0445 | 0.8689 | 1.2557 | 0.642813978 | 0.0024 | Lifestyles |
| 1458_1 | Cereal intake (medium) | 0.7999 | 0.6527 | 0.9802 | 0.031375848 | 0.9032 | Lifestyles |
| 1458_2 | Cereal intake (high) | 0.821 | 0.6996 | 0.9634 | 0.015681559 | 0.9032 | Lifestyles |
| 1498_1 | Coffee intake (medium) | 0.8494 | 0.7012 | 1.0288 | 0.095048046 | 0.7197 | Lifestyles |
| 1498_2 | Coffee intake (high) | 0.8219 | 0.696 | 0.9705 | 0.020743904 | 0.7197 | Lifestyles |
| 1528_1 | Water intake (medium) | 1.121 | 0.9449 | 1.33 | 0.190174411 | 0.5988 | Lifestyles |
| 1528_2 | Water intake (high) | 1.298 | 1.0754 | 1.5667 | 0.006576232 | 0.5988 | Lifestyles |
| 1558_1 | Alcohol intake frequency (less than once a week) | 0.8351 | 0.6211 | 1.1229 | 0.23299795 | 0.856 | Lifestyles |
| 1558_2 | Alcohol intake frequency (once a week or more) | 0.6426 | 0.4913 | 0.8406 | 0.001250889 | 0.856 | Lifestyles |
| 20160 | Ever smoked | 0.9757 | 0.8407 | 1.1323 | 0.746103883 | 0.545 | Lifestyles |
| 2139_1 | Age first had sexual intercourse (medium) | 0.854 | 0.7162 | 1.0183 | 0.078761802 | 0.8282 | Lifestyles |
| 2139_2 | Age first had sexual intercourse (late) | 0.6921 | 0.574 | 0.8345 | 0.000115807 | 0.8282 | Lifestyles |
| 2149_1 | Lifetime number of sexual partners (medium) | 1.0989 | 0.9302 | 1.2982 | 0.26734642 | 0.286 | Lifestyles |
| 2149_2 | Lifetime number of sexual partners (more) | 1.1115 | 0.9192 | 1.344 | 0.275474234 | 0.286 | Lifestyles |
| 2159 | Ever had same-sex intercourse | 0.6226 | 0.3594 | 1.0786 | 0.091038708 | 0.655 | Lifestyles |
| 22037_1 | MET minutes per week for walking (medium) | 0.9931 | 0.8287 | 1.1901 | 0.940419656 | 0.727 | Lifestyles |
| 22037_2 | MET minutes per week for walking (long) | 1.1417 | 0.9576 | 1.3612 | 0.139626173 | 0.727 | Lifestyles |
| 22038_1 | MET minutes per week for moderate activity (medium) | 0.7772 | 0.647 | 0.9335 | 0.007023152 | 0.3391 | Lifestyles |
| 22038_2 | MET minutes per week for moderate activity (long) | 0.9784 | 0.8238 | 1.1621 | 0.803891279 | 0.3391 | Lifestyles |
| 22039_1 | MET minutes per week for vigorous activity (medium) | 0.8374 | 0.7014 | 0.9997 | 0.04965699 | 0.848 | Lifestyles |
| 22039_2 | MET minutes per week for vigorous activity (long) | 0.9397 | 0.7918 | 1.1151 | 0.476171903 | 0.848 | Lifestyles |
| 6144_1 | Intake of eggs or food containing eggs | 0.4569 | 0.3254 | 0.6416 | 0.000006131 | 0.2136 | Lifestyles |
| 6144_2 | Intake of dairy product | 0.6791 | 0.4442 | 1.0381 | 0.073864747 | 0.7411 | Lifestyles |
| 6144_3 | Intake of wheat product | 0.6359 | 0.4297 | 0.9411 | 0.023585542 | 0.0614 | Lifestyles |
| 6144_4 | Intake of sugar or foods/drinks containing sugar | 0.8939 | 0.749 | 1.0669 | 0.213991895 | 0.63 | Lifestyles |
| 6155_1 | Intake of vitamin A supplements | 0.951 | 0.5702 | 1.5861 | 0.847404755 | 0.2147 | Lifestyles |
| 6155_2 | Intake of vitamin B supplements | 0.9689 | 0.668 | 1.4055 | 0.867865058 | 0.361 | Lifestyles |
| 6155_3 | Intake of vitamin C supplements | 0.9692 | 0.7527 | 1.2481 | 0.808632417 | 0.6885 | Lifestyles |
| 6155_4 | Intake of vitamin D supplements | 0.8625 | 0.594 | 1.2524 | 0.436969131 | 0.3043 | Lifestyles |
| 6155_5 | Intake of vitamin E supplements | 0.9254 | 0.6107 | 1.4023 | 0.714584672 | 0.1295 | Lifestyles |
| 6155_6 | Intake of folic acid or Folate (Vit B9) supplements | 0.8083 | 0.4669 | 1.3993 | 0.447259226 | 0.0359 | Lifestyles |
| 6155_7 | Intake of multivitamins +/- minerals supplements | 1.026 | 0.8632 | 1.2194 | 0.771178738 | 0.3314 | Lifestyles |
| 6160_1 | Join in sports club or gym | 0.7742 | 0.6583 | 0.9106 | 0.001991936 | 0.8926 | Lifestyles |
| 6160_2 | Join in pub or social club | 1.1887 | 1.0105 | 1.3983 | 0.036992411 | 0.6786 | Lifestyles |
| 6160_3 | Join in religious group | 1.0495 | 0.8709 | 1.2646 | 0.61182981 | 0.0288 | Lifestyles |
| 6160_4 | Join in adult education class | 0.7421 | 0.5586 | 0.9859 | 0.039615492 | 0.4584 | Lifestyles |
| 6160_5 | Join in other group activity | 0.8122 | 0.6825 | 0.9666 | 0.019123614 | 0.4904 | Lifestyles |
| 6179_1 | Intake of fish oil (including cod liver oil) supplements | 0.9609 | 0.8263 | 1.1175 | 0.604881134 | 0.8864 | Lifestyles |
| 6179_2 | Intake of glucosamine supplements | 0.8876 | 0.7452 | 1.0572 | 0.181307865 | 0.0192 | Lifestyles |
| 6179_3 | Intake of calcium supplements | 1.0648 | 0.8112 | 1.3977 | 0.651141793 | 0.3091 | Lifestyles |
| 6179_4 | Intake of zinc supplements | 0.9563 | 0.6673 | 1.3706 | 0.807910298 | 0.6099 | Lifestyles |
| 6179_5 | Intake of iron supplements | 1.1349 | 0.7272 | 1.7714 | 0.577341239 | 0.0886 | Lifestyles |
| 6179_6 | Intake of selenium supplements | 1.0425 | 0.6939 | 1.5662 | 0.841084646 | 0.2932 | Lifestyles |
| 24004_1 | Nitrogen oxides air pollution (medium) | 1.2105 | 1.0107 | 1.4499 | 0.037956678 | 0.9339 | Local environment |
| 24004_2 | Nitrogen oxides air pollution (high) | 1.4044 | 1.1756 | 1.6778 | 0.000181833 | 0.9339 | Local environment |
| 24005_1 | Particulate matter air pollution pm10 (medium) | 1.1199 | 0.9405 | 1.3335 | 0.203600479 | 0.6723 | Local environment |
| 24005_2 | Particulate matter air pollution pm10 (high) | 1.0483 | 0.8773 | 1.2528 | 0.603489476 | 0.6723 | Local environment |
| 24006_1 | Particulate matter air pollution pm2.5 (medium) | 1.272 | 1.064 | 1.5208 | 0.008274062 | 0.423 | Local environment |
| 24006_2 | Particulate matter air pollution pm2.5 (high) | 1.3672 | 1.142 | 1.6369 | 0.000659583 | 0.423 | Local environment |
| 24008_1 | Particulate matter air pollution 2.5-10um (medium) | 0.95 | 0.7977 | 1.1314 | 0.564999472 | 0.1774 | Local environment |
| 24008_2 | Particulate matter air pollution 2.5-10um (high) | 0.9445 | 0.7931 | 1.125 | 0.522349019 | 0.1774 | Local environment |
| 24020_1 | Average daytime sound level of noise pollution (medium) | 1.1205 | 0.9405 | 1.335 | 0.202727434 | 0.6589 | Local environment |
| 24020_2 | Average daytime sound level of noise pollution (high) | 1.0536 | 0.8814 | 1.2594 | 0.566250593 | 0.6589 | Local environment |
| 24021_1 | Average evening sound level of noise pollution (medium) | 1.1057 | 0.9281 | 1.3173 | 0.260471468 | 0.5708 | Local environment |
| 24021_2 | Average evening sound level of noise pollution (high) | 1.0473 | 0.8765 | 1.2514 | 0.611128022 | 0.5708 | Local environment |
| 24022_1 | Average night-time sound level of noise pollution (medium) | 1.1248 | 0.9443 | 1.3399 | 0.187631461 | 0.4739 | Local environment |
| 24022_2 | Average night-time sound level of noise pollution (high) | 1.0495 | 0.8778 | 1.2547 | 0.596140721 | 0.4739 | Local environment |
| 24024_1 | Average 24-hour sound level of noise pollution (medium) | 1.1235 | 0.9438 | 1.3375 | 0.190336379 | 0.5313 | Local environment |
| 24024_2 | Average 24-hour sound level of noise pollution (high) | 1.0256 | 0.8574 | 1.2268 | 0.782225354 | 0.5313 | Local environment |
| 24500_1 | Greenspace percentage, buffer 1000m (medium) | 1.129 | 0.9502 | 1.3414 | 0.167901752 | 0.1358 | Local environment |
| 24500_2 | Greenspace percentage, buffer 1000m (high) | 0.8141 | 0.6767 | 0.9794 | 0.029175953 | 0.1358 | Local environment |
| 24501_1 | Domestic garden percentage, buffer 1000m (medium) | 1.1101 | 0.9306 | 1.3243 | 0.245607169 | 0.0769 | Local environment |
| 24501_2 | Domestic garden percentage, buffer 1000m (high) | 1.0867 | 0.9096 | 1.2984 | 0.359622185 | 0.0769 | Local environment |
| 24502_1 | Water percentage, buffer 1000m (medium) | 1.028 | 0.8614 | 1.2267 | 0.759743712 | 0.4144 | Local environment |
| 24502_2 | Water percentage, buffer 1000m (high) | 1.0071 | 0.8433 | 1.2027 | 0.937453124 | 0.4144 | Local environment |
| 24506_1 | Natural environment percentage, buffer 1000m (medium) | 1.0481 | 0.8784 | 1.2506 | 0.602083664 | 0.0055 | Local environment |
| 24506_2 | Natural environment percentage, buffer 1000m (high) | 0.9482 | 0.7926 | 1.1344 | 0.561092412 | 0.0055 | Local environment |
| 130176 | Varicella | 0.1307 | 0.0738 | 0.2316 | 3.12E-12 | 0.4285 | Medical history |
| 130178 | Zoster | 1.2603 | 0.8082 | 1.9653 | 0.307401873 | 0.1457 | Medical history |
| 130184 | Measles | 0.9668 | 0.6885 | 1.3574 | 0.845167777 | 0.3693 | Medical history |
| 130186 | Rubella | 0.234 | 0.097 | 0.5642 | 0.001219098 | 0.9579 | Medical history |
| 130188 | Viral warts | 0.8075 | 0.4759 | 1.3703 | 0.428169366 | 0.6073 | Medical history |
| 130216 | Mumps | 0.444 | 0.2565 | 0.7684 | 0.003719089 | 0.8904 | Medical history |
| 130226 | Dermatophytosis | 0.7826 | 0.5252 | 1.166 | 0.228203081 | 0.5998 | Medical history |
| 130230 | Candidiasis | 0.6066 | 0.2271 | 1.6206 | 0.318761295 | 0.2978 | Medical history |
| 130622 | Iron deficiency anaemia | 0.8342 | 0.482 | 1.4436 | 0.517046563 | 0.6169 | Medical history |
| 130814 | Disorders of lipoprotein metabolism and other lipidaemias | 0.7635 | 0.6197 | 0.9407 | 0.011276308 | 0.0297 | Medical history |
| 130820 | Disorders of mineral metabolism | 2.269 | 0.8493 | 6.062 | 0.102209409 | 0.7216 | Medical history |
| 130854 | Mental and behavioural disorders due to use of alcohol | 1.5643 | 0.743 | 3.2934 | 0.238808412 | 0.472 | Medical history |
| 130868 | Mental and behavioural disorders due to use of tobacco | 1.4141 | 0.9918 | 2.0164 | 0.055556223 | 0.8521 | Medical history |
| 130894 | Depressive episode | 1.1872 | 0.922 | 1.5288 | 0.18344103 | 0.8482 | Medical history |
| 130910 | Reaction to severe stress, and adjustment disorders | 0.8679 | 0.5016 | 1.502 | 0.612756187 | 0.7258 | Medical history |
| 131052 | Migraine | 1.059 | 0.7506 | 1.4941 | 0.744239039 | 0.7833 | Medical history |
| 131056 | Transient cerebral ischaemic attacks and related syndromes | 1.1213 | 0.5581 | 2.2532 | 0.747723451 | 0.561 | Medical history |
| 131060 | Sleep disorders | 1.5173 | 0.9832 | 2.3416 | 0.05965018 | 0.1494 | Medical history |
| 131074 | Mononeuropathies of upper limb | 1.0641 | 0.6895 | 1.6422 | 0.779022519 | 0.7258 | Medical history |
| 131128 | Hordeolum and chalazion | 0.8024 | 0.4158 | 1.5484 | 0.511656881 | 0.3164 | Medical history |
| 131136 | Disorders of lachrymal system | 1.0728 | 0.5915 | 1.9459 | 0.817023931 | 0.6074 | Medical history |
| 131142 | Conjunctivitis | 0.6224 | 0.3791 | 1.0217 | 0.06076301 | 0.9433 | Medical history |
| 131164 | Senile cataract | 0.3923 | 0.0979 | 1.5719 | 0.1863759 | 0.9076 | Medical history |
| 131186 | Glaucoma | 1.0212 | 0.563 | 1.8521 | 0.945003544 | 0.1761 | Medical history |
| 131190 | Disorders of vitreous body | 0.8344 | 0.3736 | 1.8636 | 0.658831018 | 0.2868 | Medical history |
| 131208 | Disorders of refraction and accommodation | 1.0369 | 0.3881 | 2.7701 | 0.942379821 | 0.8465 | Medical history |
| 131210 | Visual disturbances | 0.8612 | 0.4091 | 1.8128 | 0.693912537 | 0.3982 | Medical history |
| 131222 | Otitis externa | 1.1546 | 0.7555 | 1.7647 | 0.506468349 | 0.2027 | Medical history |
| 131252 | Disorders of vestibular function | 1.0853 | 0.5624 | 2.0942 | 0.807247198 | 0.395 | Medical history |
| 131262 | Otalgia and effusion of ear | 0.7661 | 0.4224 | 1.3895 | 0.380410962 | 0.5793 | Medical history |
| 131286 | Essential (primary) hypertension | 1.1049 | 0.9436 | 1.2937 | 0.215218516 | 0.4508 | Medical history |
| 131296 | Angina pectoris | 1.3708 | 1.0003 | 1.8786 | 0.049809693 | 0.1483 | Medical history |
| 131298 | Acute myocardial infarction | 1.6405 | 1.1518 | 2.3363 | 0.006078566 | 0.5054 | Medical history |
| 131306 | Chronic ischaemic heart disease | 1.5028 | 1.0752 | 2.1006 | 0.017112947 | 0.8498 | Medical history |
| 131308 | Pulmonary embolism | 1.2658 | 0.6306 | 2.5407 | 0.507289065 | 0.4222 | Medical history |
| 131350 | Atrial fibrillation and flutter | 0.5855 | 0.2917 | 1.1753 | 0.132142568 | 0.4348 | Medical history |
| 131354 | Heart failure | 1.5553 | 0.6964 | 3.4736 | 0.281285108 | 0.5699 | Medical history |
| 131366 | Cerebral infarction | 0.9064 | 0.2263 | 3.6312 | 0.889656192 | 0.7646 | Medical history |
| 131396 | Phlebitis and thrombophlebitis | 0.6695 | 0.3785 | 1.1844 | 0.168042702 | 0.0782 | Medical history |
| 131402 | Varicose veins of lower extremities | 0.9548 | 0.6304 | 1.4462 | 0.82704008 | 0.9706 | Medical history |
| 131404 | Haemorrhoids | 0.9396 | 0.6088 | 1.4502 | 0.778439732 | 0.0788 | Medical history |
| 131416 | Hypotension | 1.4797 | 0.614 | 3.5657 | 0.382568612 | 0.614 | Medical history |
| 131426 | Acute sinusitis | 0.6369 | 0.3937 | 1.0302 | 0.065975893 | 0.4318 | Medical history |
| 131428 | Acute pharyngitis | 0.7934 | 0.5088 | 1.2372 | 0.307161925 | 0.9526 | Medical history |
| 131430 | Acute tonsillitis | 1.0801 | 0.7619 | 1.5313 | 0.665170692 | 0.0306 | Medical history |
| 131442 | Influenza | 0.5245 | 0.2349 | 1.1714 | 0.115465492 | 0.6816 | Medical history |
| 131456 | Pneumonia | 1.1666 | 0.7633 | 1.7831 | 0.476393794 | 0.7017 | Medical history |
| 131464 | Vasomotor and allergic rhinitis | 1.1773 | 0.9311 | 1.4886 | 0.172611809 | 0.5776 | Medical history |
| 131468 | Chronic sinusitis | 0.6944 | 0.372 | 1.296 | 0.251949897 | 0.3648 | Medical history |
| 131484 | Bronchitis | 0.6882 | 0.3566 | 1.3279 | 0.265152297 | 0.3485 | Medical history |
| 131494 | Asthma | 0.9566 | 0.7614 | 1.2018 | 0.703025047 | 0.2915 | Medical history |
| 131582 | Oesophagitis | 1.118 | 0.6588 | 1.8972 | 0.679340125 | 0.3707 | Medical history |
| 131584 | Gastro-oesophageal reflux disease | 0.9862 | 0.7458 | 1.304 | 0.922215263 | 0.646 | Medical history |
| 131590 | Gastric ulcer | 1.0379 | 0.5561 | 1.9372 | 0.906919311 | 0.6606 | Medical history |
| 131592 | Duodenal ulcer | 1.1529 | 0.6356 | 2.0911 | 0.639602392 | 0.6396 | Medical history |
| 131598 | Gastritis and duodenitis | 0.8747 | 0.607 | 1.2604 | 0.47249321 | 0.1616 | Medical history |
| 131600 | Dyspepsia | 1.2114 | 0.7589 | 1.9338 | 0.421482199 | 0.0286 | Medical history |
| 131612 | Inguinal hernia | 0.7723 | 0.5129 | 1.1628 | 0.215880586 | 0.9416 | Medical history |
| 131620 | Diaphragmatic hernia | 0.8206 | 0.566 | 1.1895 | 0.296540876 | 0.1051 | Medical history |
| 131634 | Paralytic ileus and intestinal obstruction without hernia | 0.5347 | 0.1335 | 2.142 | 0.376590769 | 0.7469 | Medical history |
| 131636 | Diverticular disease of intestine | 0.7481 | 0.4934 | 1.1344 | 0.171830691 | 0.96 | Medical history |
| 131638 | Irritable bowel syndrome | 1.1816 | 0.874 | 1.5973 | 0.278046164 | 0.5789 | Medical history |
| 131674 | Cholelithiasis | 0.7817 | 0.4957 | 1.2329 | 0.289456012 | 0.062 | Medical history |
| 131700 | Cutaneous abscess, furuncle and carbuncle | 1.6251 | 0.9903 | 2.667 | 0.054692294 | 0.1349 | Medical history |
| 131702 | Cellulitis | 1.0359 | 0.6712 | 1.5986 | 0.873550282 | 0.4507 | Medical history |
| 131720 | Atopic dermatitis | 1.0085 | 0.6049 | 1.6816 | 0.973976292 | 0.0185 | Medical history |
| 131722 | Seborrhoeic dermatitis | 1.3637 | 0.788 | 2.3599 | 0.26758772 | 0.845 | Medical history |
| 131738 | Pruritus | 1.2893 | 0.745 | 2.2311 | 0.363903847 | 0.1384 | Medical history |
| 131742 | Psoriasis | 0.9734 | 0.5931 | 1.5975 | 0.915092901 | 0.5207 | Medical history |
| 131754 | Urticaria | 0.7445 | 0.3709 | 1.4943 | 0.406505211 | 0.2456 | Medical history |
| 131794 | Follicular cysts of skin and subcutaneous tissue | 1.2324 | 0.7619 | 1.9935 | 0.394353813 | 0.4149 | Medical history |
| 131806 | Seborrhoeic keratosis | 1.4852 | 1.0246 | 2.153 | 0.036774634 | 0.5172 | Medical history |
| 131858 | Gout | 1.4647 | 0.9749 | 2.2006 | 0.066101333 | 0.1523 | Medical history |
| 131868 | Polyarthrosis | 0.7245 | 0.2712 | 1.9357 | 0.520453424 | 0.478 | Medical history |
| 131870 | Coxarthrosis | 1.1846 | 0.6844 | 2.0501 | 0.545077441 | 0.1656 | Medical history |
| 131872 | Gonarthrosis | 1.1188 | 0.7449 | 1.6804 | 0.588587282 | 0.5704 | Medical history |
| 131878 | Acquired deformities of fingers and toes | 1.1347 | 0.7194 | 1.7897 | 0.586870519 | 0.2622 | Medical history |
| 131916 | Spondylosis | 1.2624 | 0.8852 | 1.8005 | 0.198244408 | 0.3901 | Medical history |
| 131928 | Dorsalgia | 0.8797 | 0.6783 | 1.1409 | 0.333790555 | 0.283 | Medical history |
| 131938 | Synovitis and tenosynovitis | 0.6123 | 0.3173 | 1.1815 | 0.143523193 | 0.1828 | Medical history |
| 131946 | Soft tissue disorders related to use, overuse and pressure | 1.2477 | 0.7483 | 2.0804 | 0.396215526 | 0.2384 | Medical history |
| 131950 | Fibroblastic disorders | 0.8521 | 0.5264 | 1.3795 | 0.515001223 | 0.5453 | Medical history |
| 131964 | Osteoporosis without pathological fracture | 1.3241 | 0.8394 | 2.0887 | 0.22741194 | 0.2234 | Medical history |
| 132032 | Chronic renal failure | 1.0412 | 0.5396 | 2.0094 | 0.904106232 | 0.747 | Medical history |
| 132036 | Calculus of kidney and ureter | 1.1015 | 0.6226 | 1.9487 | 0.73987465 | 0.8691 | Medical history |
| 132054 | Cystitis | 0.8528 | 0.5115 | 1.4219 | 0.541554355 | 0.795 | Medical history |
| 23099_1 | Body fat percentage (medium) | 0.9812 | 0.8147 | 1.1819 | 0.841931216 | 0.7278 | Physical measures |
| 23099_2 | Body fat percentage (high) | 1.1682 | 0.9154 | 1.491 | 0.211472638 | 0.7278 | Physical measures |
| 23105_1 | Basal metabolic rate (medium) | 1.0483 | 0.8418 | 1.3055 | 0.673472159 | 0.0966 | Physical measures |
| 23105_2 | Basal metabolic rate (high) | 1.0967 | 0.8223 | 1.4628 | 0.529844563 | 0.0966 | Physical measures |
| 23111_23115_1 | Leg fat percentage (medium) | 1.0675 | 0.8641 | 1.3189 | 0.544726898 | 0.8425 | Physical measures |
| 23111_23115_2 | Leg fat percentage (high) | 1.3753 | 1.0105 | 1.8718 | 0.04271649 | 0.8425 | Physical measures |
| 23119_23123_1 | Arm fat percentage (medium) | 1.0189 | 0.8463 | 1.2267 | 0.843535506 | 0.6599 | Physical measures |
| 23119_23123_2 | Arm fat percentage (high) | 1.1044 | 0.861 | 1.4166 | 0.434243286 | 0.6599 | Physical measures |
| 23127_1 | Trunk fat percentage (medium) | 0.9706 | 0.8123 | 1.1597 | 0.74230723 | 0.0308 | Physical measures |
| 23127_2 | Trunk fat percentage (high) | 1.1189 | 0.9224 | 1.3572 | 0.254108353 | 0.0308 | Physical measures |
| 3062_1 | Forced vital capacity (medium) | 0.8334 | 0.6867 | 1.0114 | 0.065043861 | 0.5499 | Physical measures |
| 3062_2 | Forced vital capacity (high) | 0.6916 | 0.546 | 0.8762 | 0.002249318 | 0.5499 | Physical measures |
| 3063_1 | Forced expiratory volume in 1-second (medium) | 0.7674 | 0.6361 | 0.9258 | 0.005683489 | 0.7139 | Physical measures |
| 3063_2 | Forced expiratory volume in 1-second (high) | 0.6255 | 0.4982 | 0.7854 | 0.000053259 | 0.7139 | Physical measures |
| 3064_1 | Peak expiratory flow (medium) | 0.816 | 0.684 | 0.9733 | 0.023784698 | 0.5006 | Physical measures |
| 3064_2 | Peak expiratory flow (high) | 0.5344 | 0.4311 | 0.6625 | 0.000000011 | 0.5006 | Physical measures |
| 4079_1 | Diastolic blood pressure (medium) | 1.0531 | 0.8826 | 1.2565 | 0.566169707 | 0.797 | Physical measures |
| 4079_2 | Diastolic blood pressure (high) | 1.0137 | 0.8452 | 1.2158 | 0.883417363 | 0.797 | Physical measures |
| 4080_1 | Systolic blood pressure (medium) | 1.0028 | 0.8274 | 1.2154 | 0.977407311 | 0.7803 | Physical measures |
| 4080_2 | Systolic blood pressure (high) | 1.0101 | 0.8361 | 1.2203 | 0.917358041 | 0.7803 | Physical measures |
| 46_47_1 | Hand grip strength (medium) | 0.709 | 0.5753 | 0.8738 | 0.001258258 | 0.303 | Physical measures |
| 46_47_2 | Hand grip strength (high) | 0.5184 | 0.3996 | 0.6724 | 0.000000744 | 0.303 | Physical measures |
| 1920 | Mood swings | 1.2452 | 1.0757 | 1.4413 | 0.003299066 | 0.7634 | Psychosocial factors |
| 1930 | Miserableness | 1.0812 | 0.9306 | 1.2561 | 0.307538093 | 0.7637 | Psychosocial factors |
| 1940 | Irritability | 1.0058 | 0.8526 | 1.1865 | 0.945335997 | 0.6821 | Psychosocial factors |
| 1950 | Sensitivity / hurt feelings | 0.9513 | 0.8222 | 1.1006 | 0.502001538 | 0.4764 | Psychosocial factors |
| 1960 | Fed-up feelings | 1.1583 | 0.9956 | 1.3477 | 0.057108733 | 0.6319 | Psychosocial factors |
| 1970 | Nervous feelings | 0.9578 | 0.7978 | 1.1497 | 0.643303175 | 0.0682 | Psychosocial factors |
| 1980 | Worrier / anxious feelings | 1.0021 | 0.8661 | 1.1594 | 0.977858545 | 0.9636 | Psychosocial factors |
| 1990 | Tense / 'highly strung' | 1.1062 | 0.907 | 1.3492 | 0.319117664 | 0.1068 | Psychosocial factors |
| 2000 | Worry too long after embarrassment | 0.8763 | 0.757 | 1.0143 | 0.076809556 | 0.3559 | Psychosocial factors |
| 2010 | Suffer from 'nerves' | 1.0046 | 0.8305 | 1.2151 | 0.962662123 | 0.0367 | Psychosocial factors |
| 2020 | Loneliness, isolation | 1.408 | 1.1675 | 1.6981 | 0.000343675 | 0.093 | Psychosocial factors |
| 2030 | Guilty feelings | 0.9294 | 0.7846 | 1.1009 | 0.396867758 | 0.5513 | Psychosocial factors |
| 22189_1 | Townsend deprivation index at recruitment (medium) | 1.0869 | 0.9081 | 1.3009 | 0.363449155 | 0.7145 | SES |
| 22189_2 | Townsend deprivation index at recruitment (high) | 1.4091 | 1.1823 | 1.6795 | 0.000127834 | 0.7145 | SES |
| 6138 | Lower education | 1.6168 | 1.3984 | 1.8694 | 8.68E-11 | 0.3283 | SES |
| 6142 | Unemployed | 2.1195 | 1.5834 | 2.8372 | 0.000000446 | 0.3232 | SES |
| 738_1 | Average total household income before tax (18,000 to 30,999) | 0.7677 | 0.6361 | 0.9266 | 0.005877835 | 0.5214 | SES |
| 738_2 | Average total household income before tax (31,000 to 51,999) | 0.6227 | 0.5068 | 0.765 | 0.000006493 | 0.5214 | SES |
| 738_3 | Average total household income before tax (Greater than 52,000) | 0.5085 | 0.4027 | 0.642 | 0.000000013 | 0.5214 | SES |

**Table S6. Multicollinearity analysis for 45 variables.**

| Variable ID | GVIF | DF | GVIF^(1/(2*DF)) |
| --- | --- | --- | --- |
| 1210 | 1.085807 | 1 | 1.042021 |
| 1920 | 1.110431 | 1 | 1.05377 |
| 2020 | 1.118983 | 1 | 1.05782 |
| 6138 | 1.219604 | 1 | 1.104357 |
| 6142 | 1.048579 | 1 | 1.024001 |
| 130176 | 1.218983 | 1 | 1.104076 |
| 130186 | 1.185557 | 1 | 1.088833 |
| 130216 | 1.028734 | 1 | 1.014265 |
| 130814 | 1.088551 | 1 | 1.043337 |
| 131296 | 1.224707 | 1 | 1.106665 |
| 131298 | 1.059851 | 1 | 1.029491 |
| 131306 | 1.213435 | 1 | 1.10156 |
| 131806 | 1.00018 | 1 | 1.00009 |
| 6144_1 | 1.059198 | 1 | 1.029173 |
| 6144_3 | 1.048709 | 1 | 1.024065 |
| 6160_1 | 1.250224 | 1 | 1.118134 |
| 6160_2 | 1.12807 | 1 | 1.062107 |
| 6160_4 | 1.032971 | 1 | 1.016352 |
| 6160_5 | 1.052679 | 1 | 1.026001 |
| 1050 | 1.599126 | 2 | 1.124529 |
| 1060 | 1.631183 | 2 | 1.130123 |
| 1070 | 1.187999 | 2 | 1.044008 |
| 1080 | 1.074742 | 2 | 1.018183 |
| 1329 | 1.143019 | 2 | 1.033983 |
| 1349 | 1.860238 | 2 | 1.167864 |
| 1369 | 2.260695 | 2 | 1.226198 |
| 1379 | 2.09827 | 2 | 1.203553 |
| 1389 | 2.048661 | 2 | 1.196375 |
| 1458 | 1.06211 | 2 | 1.015178 |
| 1498 | 1.039397 | 2 | 1.009707 |
| 1528 | 1.086644 | 2 | 1.020991 |
| 1558 | 1.193519 | 2 | 1.045219 |
| 2139 | 1.10294 | 2 | 1.024797 |
| 3062 | 6.939139 | 2 | 1.623029 |
| 3063 | 7.237008 | 2 | 1.640173 |
| 3064 | 2.354241 | 2 | 1.23869 |
| 22038 | 1.374117 | 2 | 1.082695 |
| 22039 | 1.516232 | 2 | 1.109664 |
| 22189 | 1.298244 | 2 | 1.067429 |
| 24004 | 2.803844 | 2 | 1.294012 |
| 24006 | 2.85434 | 2 | 1.2998 |
| 24500 | 1.76862 | 2 | 1.153211 |
| 23111_23115 | 3.572274 | 2 | 1.374789 |
| 46_47 | 2.73122 | 2 | 1.285551 |
| 738 | 1.329577 | 3 | 1.048622 |

GVIF, generalized variance inflation factor; DF, degrees of freedom.

**Table S7. Associations between 184 risk factors and ALS by EWAS in subgroups.**

|  | Whole sample | | Age ≥ 65 | | Age ＜ 65 | | Male | | Female | |
| --- | --- | --- | --- | --- | --- | --- | --- | --- | --- | --- |
| Variable ID | HR | *P* value | HR | *P* value | HR | *P* value | HR | *P* value | HR | *P* value |
| 1687_1 | 1.0548 | 0.513702747 | 1.2152 | 0.135859785 | 0.9615 | 0.708396576 | 1.068 | 0.539936342 | 1.0331 | 0.796081516 |
| 1687_2 | 1.1546 | 0.174058998 | 0.8391 | 0.420526146 | 1.2892 | 0.037736325 | 1.0668 | 0.674533817 | 1.2396 | 0.141560770 |
| 1697_1 | 0.9337 | 0.486824210 | 0.9683 | 0.842793937 | 0.9137 | 0.466587575 | 0.8974 | 0.427913504 | 0.9769 | 0.869836868 |
| 1697_2 | 1.0226 | 0.796293973 | 0.9447 | 0.702386974 | 1.066 | 0.547702773 | 1.0364 | 0.757391224 | 1.0053 | 0.967464996 |
| 1787 | 1.0212 | 0.801445307 | 1.0161 | 0.915254861 | 1.0367 | 0.719529282 | 1.0055 | 0.960884805 | 1.043 | 0.736037802 |
| 1050_1 | 1.0459 | 0.631672277 | 1.0356 | 0.840849850 | 1.0508 | 0.655983298 | 1.1111 | 0.423154695 | 0.9821 | 0.892325502 |
| 1050_2 | 1.2144 | 0.037331898 | 1.2296 | 0.214921221 | 1.1984 | 0.113276542 | 1.2963 | 0.040182913 | 1.127 | 0.391835348 |
| 1060_1 | 1.1021 | 0.280879352 | 1.2466 | 0.147355653 | 1.0255 | 0.824354213 | 1.2778 | 0.053794732 | 0.9571 | 0.734674814 |
| 1060_2 | 1.395 | 0.000213584 | 1.4783 | 0.009534814 | 1.3546 | 0.007298007 | 1.7195 | 0.000003412 | 1.0147 | 0.924063775 |
| 1070_1 | 1.1426 | 0.155173868 | 1.1862 | 0.286792331 | 1.1231 | 0.316175189 | 1.0926 | 0.485031995 | 1.2023 | 0.186431811 |
| 1070_2 | 1.4421 | 0.000019485 | 1.4763 | 0.006240134 | 1.428 | 0.000956720 | 1.4242 | 0.002119328 | 1.4585 | 0.003314014 |
| 1080_1 | 1.3731 | 0.000343532 | 2.2533 | 0.000000141 | 1.045 | 0.689022694 | 1.5657 | 0.000232653 | 1.1959 | 0.161880697 |
| 1080_2 | 1.0855 | 0.409285179 | 0.9801 | 0.913667567 | 1.1481 | 0.241094782 | 1.1252 | 0.355648269 | 1.0662 | 0.688841740 |
| 1130_1 | 1.0075 | 0.958117494 | 0.6116 | 0.172865090 | 1.1467 | 0.382849599 | 0.7681 | 0.150747557 | 1.7312 | 0.013863483 |
| 1130_2 | 1.1322 | 0.410161067 | 0.7915 | 0.543037770 | 1.2435 | 0.187550163 | 1.1035 | 0.564924361 | 1.0973 | 0.773770398 |
| 1190_1 | 1.0078 | 0.920175435 | 0.9203 | 0.522707152 | 1.0527 | 0.595821882 | 1.0757 | 0.483135528 | 0.9242 | 0.505953683 |
| 1190_2 | 1.14 | 0.389544134 | 0.9538 | 0.839807746 | 1.2834 | 0.213091852 | 0.9916 | 0.965006034 | 1.6628 | 0.039015137 |
| 1200_1 | 1.0968 | 0.328872450 | 1.3845 | 0.054878264 | 0.9824 | 0.876922521 | 1.0922 | 0.458962569 | 1.0845 | 0.606671770 |
| 1200_2 | 1.1766 | 0.116951461 | 1.3486 | 0.102841182 | 1.115 | 0.389582891 | 1.2272 | 0.125648041 | 1.1153 | 0.514005555 |
| 1210 | 0.8541 | 0.045037784 | 0.8122 | 0.122471854 | 0.8858 | 0.212038645 | 0.7902 | 0.018566521 | 0.9671 | 0.788183065 |
| 1269 | 1.3696 | 0.053089336 | 1.7936 | 0.019813194 | 1.1659 | 0.472694355 | 1.8166 | 0.002300241 | 0.8702 | 0.636472210 |
| 1279 | 1.0909 | 0.374531942 | 1.1141 | 0.522645229 | 1.0778 | 0.532828192 | 1.1294 | 0.325228411 | 1.0344 | 0.833421823 |
| 1289_1 | 1.0647 | 0.465963867 | 1.0994 | 0.505813493 | 1.0437 | 0.692432741 | 1.1042 | 0.396476957 | 1.0239 | 0.852600982 |
| 1289_2 | 1.0855 | 0.379732231 | 1.017 | 0.914796286 | 1.1294 | 0.294691508 | 1.1154 | 0.379837262 | 1.0554 | 0.703388498 |
| 1299_1 | 0.9652 | 0.699446608 | 0.7411 | 0.066509860 | 1.104 | 0.375762441 | 1.0781 | 0.537137640 | 0.8333 | 0.189814775 |
| 1299_2 | 0.9293 | 0.401052868 | 0.9152 | 0.533644403 | 0.9421 | 0.589226198 | 1.0564 | 0.645370403 | 0.8008 | 0.079829566 |
| 1309_1 | 0.8947 | 0.177178579 | 0.911 | 0.511459108 | 0.8909 | 0.254187926 | 0.8376 | 0.095825481 | 0.9911 | 0.946681647 |
| 1309_2 | 0.9441 | 0.601298642 | 0.775 | 0.612883236 | 0.9187 | 0.801128901 | 1.3382 | 0.340524118 | 0.2971 | 0.087050550 |
| 1319_1 | 0.8385 | 0.059285289 | 0.6966 | 0.025974820 | 0.9236 | 0.486137587 | 0.8284 | 0.145041431 | 0.8613 | 0.271430610 |
| 1319_2 | 0.8804 | 0.189884115 | 1.2302 | 0.450973398 | 1.2927 | 0.286458393 | 1.2812 | 0.332313687 | 1.2412 | 0.399958429 |
| 1329_1 | 0.7463 | 0.027334041 | 0.6069 | 0.040426482 | 0.8105 | 0.183145261 | 0.6229 | 0.005753317 | 0.9519 | 0.815595831 |
| 1329_2 | 0.6926 | 0.003624379 | 0.5831 | 0.017384728 | 0.74 | 0.047181226 | 0.6617 | 0.010378144 | 0.7509 | 0.159884353 |
| 1339_1 | 0.6895 | 0.052132406 | 1.2761 | 0.598997297 | 0.5787 | 0.010267080 | 0.5846 | 0.027638723 | 0.8587 | 0.622875390 |
| 1339_2 | 0.8064 | 0.238542940 | 1.3676 | 0.489611420 | 0.7084 | 0.085858108 | 0.6652 | 0.079007645 | 1.0383 | 0.899141115 |
| 1349_1 | 0.7461 | 0.027979674 | 0.9294 | 0.774503829 | 0.6866 | 0.016771060 | 0.6507 | 0.043662385 | 0.8032 | 0.199053931 |
| 1349_2 | 0.8075 | 0.091083465 | 1.0325 | 0.896618654 | 0.7289 | 0.032878924 | 0.6938 | 0.060754652 | 0.8901 | 0.483474454 |
| 1359_1 | 0.9525 | 0.799851221 | 0.8448 | 0.638476120 | 1.0337 | 0.884369707 | 0.6878 | 0.159617137 | 1.3035 | 0.337544147 |
| 1359_2 | 0.8328 | 0.275274690 | 0.8795 | 0.691553439 | 0.8153 | 0.298167864 | 0.6786 | 0.092373158 | 0.9926 | 0.975637904 |
| 1369_1 | 0.7104 | 0.004574413 | 0.5527 | 0.003269179 | 0.8106 | 0.162515130 | 0.612 | 0.005143674 | 0.8034 | 0.185415861 |
| 1369_2 | 0.7633 | 0.025707086 | 0.6681 | 0.044202370 | 0.8178 | 0.185647345 | 0.693 | 0.034875222 | 0.8104 | 0.213683185 |
| 1379_1 | 0.7872 | 0.020144186 | 0.7601 | 0.130151904 | 0.8023 | 0.078575916 | 0.7251 | 0.027421126 | 0.8483 | 0.257108117 |
| 1379_2 | 0.881 | 0.266120152 | 0.8227 | 0.316816315 | 0.9158 | 0.531856099 | 0.8395 | 0.264712240 | 0.9076 | 0.560810072 |
| 1389_1 | 0.7763 | 0.014852625 | 0.7863 | 0.200192016 | 0.7777 | 0.044217814 | 0.7389 | 0.040556015 | 0.7949 | 0.116552983 |
| 1389_2 | 0.9226 | 0.483072582 | 1.0944 | 0.649815614 | 0.8379 | 0.213631971 | 0.7775 | 0.119667643 | 1.1132 | 0.508108934 |
| 1408_1 | 0.9737 | 0.915696695 | 1.0973 | 0.830927354 | 0.9157 | 0.776144419 | 1.2006 | 0.607621550 | 0.7833 | 0.494091455 |
| 1408_2 | 0.8584 | 0.523144610 | 0.9365 | 0.874012403 | 0.8202 | 0.498967518 | 0.9582 | 0.899490064 | 0.7636 | 0.426347456 |
| 1438_1 | 0.9493 | 0.560959216 | 1.0246 | 0.871584752 | 0.9077 | 0.384977373 | 0.9972 | 0.983257042 | 0.9028 | 0.407140130 |
| 1438_2 | 1.0445 | 0.642813978 | 1.0361 | 0.823500306 | 1.0483 | 0.685951819 | 1.0621 | 0.630217274 | 1.0462 | 0.764695179 |
| 1458_1 | 0.7999 | 0.031375848 | 0.8937 | 0.545697481 | 0.7637 | 0.031061261 | 0.7286 | 0.023856055 | 0.9032 | 0.510401233 |
| 1458_2 | 0.821 | 0.015681559 | 0.9145 | 0.515527826 | 0.7732 | 0.012061327 | 0.7409 | 0.006274221 | 0.9371 | 0.596097401 |
| 1498_1 | 0.8494 | 0.095048046 | 0.83 | 0.245108858 | 0.8615 | 0.226833940 | 0.7734 | 0.054138583 | 0.945 | 0.693614570 |
| 1498_2 | 0.8219 | 0.020743904 | 0.8119 | 0.147333075 | 0.8299 | 0.076035411 | 0.7512 | 0.011497016 | 0.9123 | 0.472311553 |
| 1528_1 | 1.121 | 0.190174411 | 0.9401 | 0.660004253 | 1.2519 | 0.044738722 | 1.1738 | 0.151523361 | 1.0453 | 0.750294309 |
| 1528_2 | 1.298 | 0.006576232 | 1.0869 | 0.614375227 | 1.4347 | 0.002545370 | 1.2524 | 0.092339376 | 1.3086 | 0.056635875 |
| 1558_1 | 0.8351 | 0.232997950 | 0.7001 | 0.122471731 | 0.9555 | 0.821357412 | 0.6758 | 0.068967466 | 1.0203 | 0.925315903 |
| 1558_2 | 0.6426 | 0.001250889 | 0.5237 | 0.001605346 | 0.7505 | 0.121714415 | 0.5124 | 0.000315566 | 0.8096 | 0.294404644 |
| 20160 | 0.9757 | 0.746103883 | 1.0352 | 0.790884646 | 0.9497 | 0.581982514 | 1.0227 | 0.831500830 | 0.9318 | 0.520868970 |
| 2139_1 | 0.854 | 0.078761802 | 0.7099 | 0.030690848 | 0.9155 | 0.416776639 | 0.7082 | 0.003129321 | 1.1368 | 0.375026953 |
| 2139_2 | 0.6921 | 0.000115807 | 0.4848 | 0.000004500 | 0.8326 | 0.121861007 | 0.5186 | 0.000000189 | 1.0319 | 0.836374666 |
| 2149_1 | 1.0989 | 0.267346420 | 1.3932 | 0.017402876 | 0.956 | 0.673691703 | 1.2617 | 0.051447187 | 0.9545 | 0.704270257 |
| 2149_2 | 1.1115 | 0.275474234 | 1.434 | 0.030527120 | 0.966 | 0.770133016 | 1.1921 | 0.165216992 | 1.0483 | 0.763006610 |
| 2159 | 0.6226 | 0.091038708 | 0.7449 | 0.559461253 | 0.5741 | 0.099668275 | 0.4153 | 0.032769923 | 1.0881 | 0.825688537 |
| 22037_1 | 0.9931 | 0.940419656 | 1.3668 | 0.052915319 | 0.8405 | 0.129275583 | 1.0884 | 0.497984264 | 0.889 | 0.389858864 |
| 22037_2 | 1.1417 | 0.139626173 | 1.3753 | 0.048291127 | 1.0533 | 0.632709666 | 1.2051 | 0.123270351 | 1.0675 | 0.625544651 |
| 22038_1 | 0.7772 | 0.007023152 | 0.8804 | 0.435177507 | 0.7259 | 0.005369408 | 0.7439 | 0.021590352 | 0.8063 | 0.115774935 |
| 22038_2 | 0.9784 | 0.803891279 | 1.012 | 0.937932275 | 0.9651 | 0.741464633 | 1.0605 | 0.610026500 | 0.8785 | 0.338003818 |
| 22039_1 | 0.8374 | 0.049656990 | 0.9553 | 0.768253694 | 0.7818 | 0.027353929 | 0.8597 | 0.222472789 | 0.8104 | 0.111732801 |
| 22039_2 | 0.9397 | 0.476171903 | 1.2038 | 0.207927737 | 0.8161 | 0.062847641 | 0.9569 | 0.705180174 | 0.9124 | 0.488854350 |
| 6144_1 | 0.4569 | 0.000006131 | 0.5138 | 0.024287602 | 0.4282 | 0.000072556 | 0.4453 | 0.000305204 | 0.4744 | 0.006326442 |
| 6144_2 | 0.6791 | 0.073864747 | 0.6022 | 0.115855166 | 0.7444 | 0.312888015 | 0.6828 | 0.212015105 | 0.6759 | 0.201495534 |
| 6144_3 | 0.6359 | 0.023585542 | 1.338 | 0.519325727 | 0.4707 | 0.000750503 | 0.5332 | 0.025722457 | 0.7436 | 0.295215959 |
| 6144_4 | 0.8939 | 0.213991895 | 0.9214 | 0.572836855 | 0.8715 | 0.232577584 | 0.9361 | 0.587354550 | 0.8448 | 0.210019415 |
| 6155_1 | 0.951 | 0.847404755 | 1.0153 | 0.968360656 | 0.8966 | 0.759453315 | 1.475 | 0.184887625 | 0.3913 | 0.105821176 |
| 6155_2 | 0.9689 | 0.867865058 | 0.71 | 0.340646722 | 1.1245 | 0.599962377 | 1.2394 | 0.414637306 | 0.7846 | 0.374359476 |
| 6155_3 | 0.9692 | 0.808632417 | 0.8812 | 0.562399338 | 1.0236 | 0.884136220 | 1.0315 | 0.857091778 | 0.8994 | 0.585736868 |
| 6155_4 | 0.8625 | 0.436969131 | 0.9036 | 0.722954035 | 0.8299 | 0.465050998 | 1.0367 | 0.902289063 | 0.762 | 0.275808616 |
| 6155_5 | 0.9254 | 0.714584672 | 0.5953 | 0.209399377 | 1.1496 | 0.572925704 | 1.0922 | 0.763585834 | 0.7918 | 0.446569290 |
| 6155_6 | 0.8083 | 0.447259226 | 0.6018 | 0.313605836 | 0.9468 | 0.871141334 | 0.8537 | 0.700697209 | 0.7766 | 0.508084363 |
| 6155_7 | 1.026 | 0.771178738 | 0.9449 | 0.709316171 | 1.0707 | 0.527848961 | 0.9513 | 0.694703305 | 1.1063 | 0.411397209 |
| 6160_1 | 0.7742 | 0.001991936 | 0.8636 | 0.286644646 | 0.728 | 0.002178245 | 0.7574 | 0.013859715 | 0.7951 | 0.059665420 |
| 6160_2 | 1.1887 | 0.036992411 | 1.3977 | 0.015329189 | 1.0953 | 0.379820385 | 1.1475 | 0.182405801 | 1.2684 | 0.084260036 |
| 6160_3 | 1.0495 | 0.611829810 | 1.0275 | 0.855403894 | 1.0556 | 0.661773819 | 1.0182 | 0.896738490 | 1.0707 | 0.601194452 |
| 6160_4 | 0.7421 | 0.039615492 | 0.6217 | 0.047573354 | 0.8291 | 0.302286669 | 1.0361 | 0.863792727 | 0.5729 | 0.005637983 |
| 6160_5 | 0.8122 | 0.019123614 | 0.8573 | 0.263103094 | 0.7771 | 0.030675023 | 0.7115 | 0.011673945 | 0.9001 | 0.378926529 |
| 6179_1 | 0.9609 | 0.604881134 | 1.1019 | 0.432453793 | 0.8836 | 0.213819685 | 0.7993 | 0.037875757 | 1.1807 | 0.137243973 |
| 6179_2 | 0.8876 | 0.181307865 | 0.8222 | 0.173066724 | 0.9363 | 0.562901562 | 0.7639 | 0.043469977 | 1.0112 | 0.927086847 |
| 6179_3 | 1.0648 | 0.651141793 | 1.0085 | 0.969913188 | 1.1026 | 0.580928122 | 1.4826 | 0.112172190 | 0.9347 | 0.683686209 |
| 6179_4 | 0.9563 | 0.807910298 | 0.9047 | 0.745162353 | 0.9848 | 0.946718662 | 0.8613 | 0.596341614 | 1.0419 | 0.865527668 |
| 6179_5 | 1.1349 | 0.577341239 | 1.4932 | 0.264391500 | 0.9638 | 0.899927880 | 0.8233 | 0.636331461 | 1.3897 | 0.229302780 |
| 6179_6 | 1.0425 | 0.841084646 | 0.6836 | 0.320752622 | 1.321 | 0.259992668 | 1.0914 | 0.756520687 | 0.9927 | 0.980989476 |
| 24004_1 | 1.2105 | 0.037956678 | 1.3762 | 0.034748773 | 1.1195 | 0.331214927 | 1.1041 | 0.433771077 | 1.3389 | 0.030111068 |
| 24004_2 | 1.4044 | 0.000181833 | 1.4397 | 0.019198387 | 1.3752 | 0.004350926 | 1.557 | 0.000224795 | 1.2236 | 0.146164815 |
| 24005_1 | 1.1199 | 0.203600479 | 1.02 | 0.892844179 | 1.1813 | 0.137146647 | 1.1029 | 0.420522589 | 1.1367 | 0.327580788 |
| 24005_2 | 1.0483 | 0.603489476 | 0.9226 | 0.596100908 | 1.1245 | 0.302296659 | 1.1528 | 0.240484141 | 0.9263 | 0.578207145 |
| 24006_1 | 1.272 | 0.008274062 | 1.5456 | 0.003398926 | 1.126 | 0.305477523 | 1.1852 | 0.170524811 | 1.3785 | 0.017123215 |
| 24006_2 | 1.3672 | 0.000659583 | 1.3119 | 0.092364537 | 1.3809 | 0.003926966 | 1.4487 | 0.002350326 | 1.2639 | 0.093856407 |
| 24008_1 | 0.95 | 0.564999472 | 0.7864 | 0.109281058 | 1.0531 | 0.642331804 | 0.9229 | 0.511818647 | 0.9808 | 0.881558973 |
| 24008_2 | 0.9445 | 0.522349019 | 0.8388 | 0.233319175 | 1.0109 | 0.923021123 | 1.0467 | 0.699957647 | 0.8255 | 0.157819999 |
| 24020_1 | 1.1205 | 0.202727434 | 1.1162 | 0.453518773 | 1.124 | 0.299632181 | 1.0592 | 0.632176019 | 1.1995 | 0.173989438 |
| 24020_2 | 1.0536 | 0.566250593 | 0.9517 | 0.748106140 | 1.1124 | 0.345776400 | 1.0222 | 0.856962429 | 1.0948 | 0.510101766 |
| 24021_1 | 1.1057 | 0.260471468 | 1.071 | 0.638001796 | 1.1282 | 0.285887002 | 1.054 | 0.662111418 | 1.1705 | 0.237869242 |
| 24021_2 | 1.0473 | 0.611128022 | 0.8983 | 0.486709548 | 1.1369 | 0.255581801 | 1.0325 | 0.792046706 | 1.0662 | 0.639897904 |
| 24022_1 | 1.1248 | 0.187631461 | 1.1383 | 0.374802356 | 1.1178 | 0.323694937 | 1.074 | 0.552311677 | 1.1887 | 0.195216584 |
| 24022_2 | 1.0495 | 0.596140721 | 0.9273 | 0.626949755 | 1.1196 | 0.316850852 | 1.0303 | 0.806311531 | 1.0741 | 0.602853472 |
| 24024_1 | 1.1235 | 0.190336379 | 1.1158 | 0.451491251 | 1.1291 | 0.279913087 | 1.0583 | 0.635496702 | 1.2079 | 0.156598814 |
| 24024_2 | 1.0256 | 0.782225354 | 0.9016 | 0.505582604 | 1.0978 | 0.410196396 | 0.9903 | 0.936646956 | 1.0722 | 0.613577272 |
| 24500_1 | 1.129 | 0.167901752 | 1.3198 | 0.065433305 | 1.0405 | 0.714874659 | 1.0656 | 0.594005593 | 1.2135 | 0.137936191 |
| 24500_2 | 0.8141 | 0.029175953 | 0.8744 | 0.409153146 | 0.7908 | 0.042995080 | 0.805 | 0.084647160 | 0.8284 | 0.186740974 |
| 24501_1 | 1.1101 | 0.245607169 | 1.1593 | 0.337624280 | 1.0817 | 0.478780098 | 1.0168 | 0.890126305 | 1.2405 | 0.112983270 |
| 24501_2 | 1.0867 | 0.359622185 | 1.1985 | 0.233823998 | 1.0245 | 0.830705781 | 1.029 | 0.813311708 | 1.1651 | 0.267359004 |
| 24502_1 | 1.028 | 0.759743712 | 1.2519 | 0.127027183 | 0.9121 | 0.422089787 | 0.9527 | 0.687104530 | 1.1349 | 0.353893228 |
| 24502_2 | 1.0071 | 0.937453124 | 0.8873 | 0.453393861 | 1.0696 | 0.541249224 | 0.9085 | 0.430153641 | 1.1472 | 0.312727794 |
| 24506_1 | 1.0481 | 0.602083664 | 1.1396 | 0.387867457 | 1.0011 | 0.992044763 | 0.9054 | 0.409287588 | 1.2611 | 0.089438529 |
| 24506_2 | 0.9482 | 0.561092412 | 0.9001 | 0.502832708 | 0.9812 | 0.865910391 | 0.8232 | 0.109478081 | 1.1412 | 0.343245578 |
| 130176 | 0.1307 | 0.000000000 | 0.1363 | 0.000616327 | 0.1294 | 0.000000001 | 0.134 | 0.000000144 | 0.1272 | 0.000005056 |
| 130178 | 1.2603 | 0.307401873 | 2.119 | 0.011042459 | 0.7858 | 0.498953354 | 1.5273 | 0.132954269 | 0.953 | 0.899709215 |
| 130184 | 0.9668 | 0.845167777 | 1.3782 | 0.200836626 | 0.7559 | 0.244014305 | 1.1174 | 0.612494154 | 0.7891 | 0.402403878 |
| 130186 | 0.234 | 0.001219098 | 0.4034 | 0.201399047 | 0.1843 | 0.003518469 | 0.1014 | 0.022301407 | 0.35 | 0.037153077 |
| 130188 | 0.8075 | 0.428169366 | 1.1498 | 0.715535238 | 0.623 | 0.213965613 | 0.7295 | 0.408050445 | 0.9066 | 0.797423406 |
| 130216 | 0.444 | 0.003719089 | 0.6526 | 0.301661495 | 0.3489 | 0.005690383 | 0.4919 | 0.046975927 | 0.3847 | 0.034038352 |
| 130226 | 0.7826 | 0.228203081 | 1.2125 | 0.483055905 | 0.5387 | 0.042558846 | 1.0034 | 0.988626375 | 0.4994 | 0.069106221 |
| 130230 | 0.6066 | 0.318761295 | 1.2967 | 0.654584213 | 0.2332 | 0.145895826 | 0.586 | 0.450937868 | 0.629 | 0.513350957 |
| 130622 | 0.8342 | 0.517046563 | 1.3195 | 0.469282584 | 0.5827 | 0.188692502 | 0.6419 | 0.324512566 | 1.035 | 0.923501924 |
| 130814 | 0.7635 | 0.011276308 | 0.8163 | 0.185999054 | 0.7174 | 0.025605501 | 0.7334 | 0.020087496 | 0.8292 | 0.287055552 |
| 130820 | 2.269 | 0.102209409 | 1.4486 | 0.711501459 | 2.759 | 0.079729201 | 0 | 0.984600000 | 5.365 | 0.000839467 |
| 130854 | 1.5643 | 0.238808412 | 1.9704 | 0.242963019 | 1.3555 | 0.544675450 | 1.7139 | 0.231196248 | 1.2856 | 0.723162083 |
| 130868 | 1.4141 | 0.055556223 | 1.8977 | 0.019957988 | 1.1866 | 0.476930008 | 1.3468 | 0.205528078 | 1.5254 | 0.136117048 |
| 130894 | 1.1872 | 0.183441030 | 0.8518 | 0.534259281 | 1.3622 | 0.038674693 | 1.2002 | 0.308221775 | 1.1723 | 0.392633601 |
| 130910 | 0.8679 | 0.612756187 | 1.1537 | 0.423564924 | 0.8434 | 0.226452706 | 0.8838 | 0.420576164 | 1.035 | 0.834015607 |
| 131052 | 1.059 | 0.744239039 | 1.4124 | 0.194127055 | 0.8851 | 0.602135565 | 1.0742 | 0.772527018 | 1.0457 | 0.857575628 |
| 131056 | 1.1213 | 0.747723451 | 1.6757 | 0.178178808 | 0.3277 | 0.265145903 | 1.2956 | 0.529668905 | 0.8208 | 0.780896250 |
| 131060 | 1.5173 | 0.059650180 | 1.8185 | 0.077921090 | 1.349 | 0.305838195 | 2.0463 | 0.004991209 | 0.8284 | 0.676100593 |
| 131074 | 1.0641 | 0.779022519 | 0.8349 | 0.662149795 | 1.1961 | 0.495012912 | 0.7963 | 0.523542449 | 1.3427 | 0.297582673 |
| 131128 | 0.8024 | 0.511656881 | 0.7575 | 0.632503222 | 0.828 | 0.645862530 | 0.7815 | 0.583788632 | 0.832 | 0.714656798 |
| 131136 | 1.0728 | 0.817023931 | 1.9876 | 0.055766968 | 0.4818 | 0.207412221 | 1.1331 | 0.761292930 | 1.0074 | 0.987003435 |
| 131142 | 0.6224 | 0.060763010 | 0.4012 | 0.070046591 | 0.7591 | 0.346098910 | 0.6912 | 0.301072848 | 0.5663 | 0.112145488 |
| 131164 | 0.3923 | 0.186375900 | 0.7633 | 0.703565270 | 0 | 0.985500000 | 0 | 0.985300000 | 0.819 | 0.778491830 |
| 131186 | 1.0212 | 0.945003544 | 0.2712 | 0.192825069 | 1.412 | 0.280350147 | 1.1682 | 0.683486122 | 0.8356 | 0.721110402 |
| 131190 | 0.8344 | 0.658831018 | 1.3497 | 0.551697722 | 0.4716 | 0.288836488 | 1.311 | 0.547338056 | 0.2956 | 0.223620560 |
| 131208 | 1.0369 | 0.942379821 | 1.5584 | 0.531996207 | 0.7781 | 0.723320131 | 1.4481 | 0.522851624 | 0.5625 | 0.565628653 |
| 131210 | 0.8612 | 0.693912537 | 0.9391 | 0.913883127 | 0.8074 | 0.670063827 | 1.0623 | 0.893128530 | 0.5903 | 0.457388020 |
| 131222 | 1.1546 | 0.506468349 | 1.9174 | 0.022154211 | 0.731 | 0.351712337 | 1.348 | 0.272159031 | 0.924 | 0.825310251 |
| 131252 | 1.0853 | 0.807247198 | 1.7453 | 0.217520569 | 0.7385 | 0.546071139 | 1.3431 | 0.473190682 | 0.7816 | 0.670904388 |
| 131262 | 0.7661 | 0.380410962 | 0.9693 | 0.944945539 | 0.6521 | 0.298067044 | 0.9446 | 0.881231112 | 0.5784 | 0.276466158 |
| 131286 | 1.1049 | 0.215218516 | 0.867 | 0.275109786 | 1.2898 | 0.012002155 | 0.9813 | 0.859069035 | 1.3023 | 0.029802616 |
| 131296 | 1.3708 | 0.049809693 | 1.3105 | 0.294837346 | 1.4057 | 0.097528009 | 1.0473 | 0.844029653 | 1.8793 | 0.004242645 |
| 131298 | 1.6405 | 0.006078566 | 1.1837 | 0.556552223 | 2.1427 | 0.000994363 | 1.6381 | 0.012223607 | 1.7786 | 0.202032262 |
| 131306 | 1.5028 | 0.017112947 | 1.4933 | 0.158727269 | 1.5023 | 0.056854576 | 1.3524 | 0.210446191 | 1.6982 | 0.028869403 |
| 131308 | 1.2658 | 0.507289065 | 1.2921 | 0.658974011 | 1.2534 | 0.615359745 | 1.1594 | 0.768510616 | 1.4043 | 0.499668965 |
| 131350 | 0.5855 | 0.132142568 | 0.5327 | 0.278245965 | 0.6184 | 0.285158466 | 0.3551 | 0.073985026 | 0.9589 | 0.925748465 |
| 131354 | 1.5553 | 0.281285108 | 1.3966 | 0.638023281 | 1.6483 | 0.319598201 | 1.2582 | 0.691809362 | 2.0668 | 0.210664505 |
| 131366 | 0.9064 | 0.889656192 | 1.2255 | 0.839189349 | 0.7151 | 0.737651316 | 0 | 0.988800000 | 1.9733 | 0.337889952 |
| 131396 | 0.6695 | 0.168042702 | 1.0581 | 0.882894282 | 0.4425 | 0.069738229 | 0.6268 | 0.256019201 | 0.7195 | 0.424159642 |
| 131402 | 0.9548 | 0.827040080 | 1.1495 | 0.665655781 | 0.8449 | 0.548936956 | 0.9603 | 0.890069131 | 0.9445 | 0.852246186 |
| 131404 | 0.9396 | 0.778439732 | 0.916 | 0.807061063 | 0.9533 | 0.865089702 | 0.8965 | 0.709283153 | 1.0035 | 0.991672852 |
| 131416 | 1.4797 | 0.382568612 | 0 | 0.988419929 | 2.415 | 0.049849907 | 1.6429 | 0.391629905 | 1.2829 | 0.725420165 |
| 131426 | 0.6369 | 0.065975893 | 1.0561 | 0.865521407 | 0.4062 | 0.017993065 | 0.8694 | 0.632931556 | 0.3867 | 0.035007738 |
| 131428 | 0.7934 | 0.307161925 | 0.5711 | 0.214745970 | 0.9108 | 0.721735667 | 1.1403 | 0.617815645 | 0.4155 | 0.051302370 |
| 131430 | 1.0801 | 0.665170692 | 1.0683 | 0.830162979 | 1.091 | 0.689840083 | 1.2741 | 0.279546438 | 0.8489 | 0.577473582 |
| 131442 | 0.5245 | 0.115465492 | 0.5078 | 0.339708936 | 0.5325 | 0.209408137 | 0.4783 | 0.203122830 | 0.585 | 0.355341910 |
| 131456 | 1.1666 | 0.476393794 | 1.5641 | 0.146438058 | 0.9269 | 0.803574070 | 1.3528 | 0.266492115 | 0.938 | 0.857971619 |
| 131464 | 1.1773 | 0.172611809 | 1.2465 | 0.270371920 | 1.1405 | 0.379001943 | 1.4553 | 0.011476099 | 0.8524 | 0.434414487 |
| 131468 | 0.6944 | 0.251949897 | 1.2054 | 0.650983665 | 0.4262 | 0.089441959 | 0.8837 | 0.745788083 | 0.4634 | 0.184793366 |
| 131484 | 0.6882 | 0.265152297 | 0.8586 | 0.762269584 | 0.5955 | 0.248870894 | 0.8506 | 0.693918873 | 0.4993 | 0.231085154 |
| 131494 | 0.9566 | 0.703025047 | 1.203 | 0.302777690 | 0.8263 | 0.214009392 | 1.1919 | 0.225712556 | 0.6877 | 0.057960831 |
| 131582 | 1.118 | 0.679340125 | 1.8024 | 0.100902179 | 0.7432 | 0.470107671 | 0.977 | 0.951399541 | 1.3073 | 0.483040452 |
| 131584 | 0.9862 | 0.922215263 | 0.9031 | 0.684300190 | 1.0332 | 0.850606525 | 0.9045 | 0.614382956 | 1.0852 | 0.688885071 |
| 131590 | 1.0379 | 0.906919311 | 1.5076 | 0.363246876 | 0.7923 | 0.604587197 | 1.1369 | 0.754986379 | 0.9268 | 0.879945580 |
| 131592 | 1.1529 | 0.639602392 | 1.4277 | 0.430448644 | 0.9977 | 0.995498806 | 1.4043 | 0.341654255 | 0.7772 | 0.663885708 |
| 131598 | 0.8747 | 0.472493210 | 0.783 | 0.447969122 | 0.9306 | 0.753006757 | 0.8429 | 0.502865863 | 0.9085 | 0.725407327 |
| 131600 | 1.2114 | 0.421482199 | 0.9107 | 0.835791763 | 1.3905 | 0.241028316 | 1.0085 | 0.980994545 | 1.4473 | 0.249577306 |
| 131612 | 0.7723 | 0.215880586 | 1.0025 | 0.992668070 | 0.5836 | 0.093162436 | 0.7391 | 0.198828239 | 0.9726 | 0.950788648 |
| 131620 | 0.8206 | 0.296540876 | 0.9204 | 0.770553502 | 0.7504 | 0.258751763 | 0.8691 | 0.571382572 | 0.7603 | 0.351359189 |
| 131634 | 0.5347 | 0.376590769 | 0 | 0.987387995 | 0.9093 | 0.893274872 | 0.484 | 0.468621584 | 0.6069 | 0.618056101 |
| 131636 | 0.7481 | 0.171830691 | 0.6873 | 0.223570503 | 0.8037 | 0.455707884 | 0.9917 | 0.974060021 | 0.4747 | 0.051596592 |
| 131638 | 1.1816 | 0.278046164 | 0.9564 | 0.875560639 | 1.3081 | 0.142135609 | 1.2695 | 0.230818326 | 1.0702 | 0.779445804 |
| 131674 | 0.7817 | 0.289456012 | 1.2776 | 0.426628459 | 0.511 | 0.059747755 | 0.7437 | 0.379649326 | 0.8176 | 0.530456131 |
| 131700 | 1.6251 | 0.054692294 | 1.4753 | 0.389145512 | 1.7098 | 0.078682476 | 2.081 | 0.016497564 | 1.097 | 0.837157562 |
| 131702 | 1.0359 | 0.873550282 | 0.5233 | 0.198681641 | 1.347 | 0.227779276 | 1.0257 | 0.930966315 | 1.046 | 0.894118816 |
| 131720 | 1.0085 | 0.973976292 | 1.2559 | 0.552022263 | 0.8567 | 0.664490729 | 1.3591 | 0.315422780 | 0.5904 | 0.294791299 |
| 131722 | 1.3637 | 0.267587720 | 1.7327 | 0.183199929 | 1.1488 | 0.715572977 | 1.3504 | 0.430677720 | 1.3803 | 0.434085577 |
| 131738 | 1.2893 | 0.363903847 | 1.7526 | 0.174314092 | 1.0585 | 0.881300524 | 1.5438 | 0.223889479 | 1.0183 | 0.967864179 |
| 131742 | 0.9734 | 0.915092901 | 0.8605 | 0.739331692 | 1.0338 | 0.913145015 | 0.7383 | 0.426034311 | 1.2997 | 0.437953710 |
| 131754 | 0.7445 | 0.406505211 | 0 | 0.987283691 | 1.1409 | 0.711679919 | 0.907 | 0.828171335 | 0.5696 | 0.331787861 |
| 131794 | 1.2324 | 0.394353813 | 1.2596 | 0.576233749 | 1.2249 | 0.505973300 | 1.0511 | 0.889055104 | 1.4599 | 0.262832046 |
| 131806 | 1.4852 | 0.036774634 | 1.2648 | 0.488743556 | 1.6032 | 0.038798347 | 1.5594 | 0.072897775 | 1.3946 | 0.257989661 |
| 131858 | 1.4647 | 0.066101333 | 0.968 | 0.937291187 | 1.7695 | 0.017638390 | 1.3842 | 0.216511374 | 1.6195 | 0.153653479 |
| 131868 | 0.7245 | 0.520453424 | 0 | 0.990324498 | 1.1975 | 0.719654018 | 0.3538 | 0.299451151 | 1.1062 | 0.861804940 |
| 131870 | 1.1846 | 0.545077441 | 1.2796 | 0.550581728 | 1.1041 | 0.794813687 | 1.5678 | 0.182241727 | 0.7528 | 0.572514663 |
| 131872 | 1.1188 | 0.588587282 | 1.1961 | 0.578630176 | 1.0672 | 0.810452325 | 1.5046 | 0.090209316 | 0.6304 | 0.262717745 |
| 131878 | 1.1347 | 0.586870519 | 1.0908 | 0.820700224 | 1.1604 | 0.611020792 | 1.2808 | 0.439546447 | 1.0024 | 0.994358359 |
| 131916 | 1.2624 | 0.198244408 | 0.8309 | 0.227067988 | 0.9055 | 0.428824222 | 0.7542 | 0.050177515 | 1.011 | 0.934986415 |
| 131928 | 0.8797 | 0.333790555 | 0.8015 | 0.341541621 | 0.9248 | 0.628359980 | 0.874 | 0.458032926 | 0.8873 | 0.538502963 |
| 131938 | 0.6123 | 0.143523193 | 0.917 | 0.847807254 | 0.4336 | 0.096072938 | 0.516 | 0.187952422 | 0.7184 | 0.463084460 |
| 131946 | 1.2477 | 0.396215526 | 1.8475 | 0.087383313 | 0.9052 | 0.793661889 | 1.6798 | 0.089704219 | 0.7322 | 0.535453651 |
| 131950 | 0.8521 | 0.515001223 | 0.7532 | 0.459928906 | 0.9337 | 0.830215844 | 1.0512 | 0.854519599 | 0.4618 | 0.182951617 |
| 131964 | 1.3241 | 0.227411940 | 0.8849 | 0.786707054 | 1.6066 | 0.080597336 | 1.234 | 0.555934270 | 1.3806 | 0.293170101 |
| 132032 | 1.0412 | 0.904106232 | 0.5435 | 0.390297966 | 1.3998 | 0.377105149 | 1.2353 | 0.607401501 | 0.7953 | 0.692939107 |
| 132036 | 1.1015 | 0.739874650 | 0.256 | 0.173840794 | 1.5804 | 0.133587591 | 1.0352 | 0.927641664 | 1.2149 | 0.665774594 |
| 132054 | 0.8528 | 0.541554355 | 0.8112 | 0.643097354 | 0.8791 | 0.686918430 | 1.1468 | 0.653964921 | 0.5004 | 0.168765284 |
| 23099_1 | 0.9812 | 0.841931216 | 0.9435 | 0.708808901 | 1.0077 | 0.949146913 | 0.9248 | 0.467314013 | 1.1513 | 0.556711253 |
| 23099_2 | 1.1682 | 0.211472638 | 1.0787 | 0.723872514 | 1.2279 | 0.180236249 | 1.5345 | 0.045238972 | 1.223 | 0.380966205 |
| 23105_1 | 1.0483 | 0.673472159 | 1.0738 | 0.720062377 | 1.0334 | 0.808552626 | 0.4821 | 0.080872762 | 1.1294 | 0.287959310 |
| 23105_2 | 1.0967 | 0.529844563 | 0.9422 | 0.810430836 | 1.2337 | 0.254591190 | 0.5256 | 0.119529740 | 0.6929 | 0.468000802 |
| 23111_23115_1 | 1.0675 | 0.544726898 | 1.0819 | 0.645858949 | 1.0625 | 0.663067196 | 1.0547 | 0.630049318 | 0.8637 | 0.837441013 |
| 23111_23115_2 | 1.3753 | 0.042716490 | 1.4959 | 0.140945906 | 1.3404 | 0.132201540 | 2.6481 | 0.030861925 | 1.0668 | 0.927411824 |
| 23119_23123_1 | 1.0189 | 0.843535506 | 0.8855 | 0.434647667 | 1.1111 | 0.378090393 | 0.9452 | 0.599284508 | 1.348 | 0.228318794 |
| 23119_23123_2 | 1.1044 | 0.434243286 | 0.8153 | 0.349511729 | 1.3019 | 0.092193438 | 1.4015 | 0.189979702 | 1.327 | 0.238823421 |
| 23127_1 | 0.9706 | 0.742307230 | 0.9851 | 0.920260030 | 0.9617 | 0.732714216 | 0.9744 | 0.812928627 | 0.9519 | 0.764125210 |
| 23127_2 | 1.1189 | 0.254108353 | 0.9382 | 0.705105957 | 1.2381 | 0.078932299 | 1.1908 | 0.212746909 | 1.0561 | 0.714039943 |
| 3062_1 | 0.8334 | 0.065043861 | 0.7466 | 0.084430134 | 0.8866 | 0.326005974 | 0.7124 | 0.034391864 | 0.9432 | 0.636451791 |
| 3062_2 | 0.6916 | 0.002249318 | 0.6032 | 0.011046828 | 0.7515 | 0.060272877 | 0.6099 | 0.001277122 | 0.7094 | 0.321608318 |
| 3063_1 | 0.7674 | 0.005683489 | 0.7672 | 0.110256258 | 0.7673 | 0.025221097 | 0.5655 | 0.000053375 | 0.998 | 0.986935079 |
| 3063_2 | 0.6255 | 0.000053259 | 0.6973 | 0.062378501 | 0.5909 | 0.000281414 | 0.4956 | 0.000000149 | 0.8714 | 0.653373617 |
| 3064_1 | 0.816 | 0.023784698 | 0.7119 | 0.025046265 | 0.887 | 0.288439924 | 0.7837 | 0.073413333 | 0.8583 | 0.204915226 |
| 3064_2 | 0.5344 | 0.000000011 | 0.42 | 0.000003720 | 0.6112 | 0.000320359 | 0.4984 | 0.000000081 | 0.7289 | 0.261294231 |
| 4079_1 | 1.0531 | 0.566169707 | 1.1495 | 0.343984104 | 1.0093 | 0.935102671 | 0.892 | 0.360929573 | 1.2389 | 0.096709217 |
| 4079_2 | 1.0137 | 0.883417363 | 0.9545 | 0.768674359 | 1.0562 | 0.633872626 | 0.8675 | 0.248053726 | 1.1977 | 0.195997538 |
| 4080_1 | 1.0028 | 0.977407311 | 1.1286 | 0.516945717 | 0.9609 | 0.731607763 | 0.9346 | 0.627067414 | 1.0615 | 0.665787875 |
| 4080_2 | 1.0101 | 0.917358041 | 1.0337 | 0.850711746 | 1.0182 | 0.877567888 | 0.9935 | 0.961042297 | 0.9973 | 0.984900062 |
| 46_47_1 | 0.709 | 0.001258258 | 0.6738 | 0.041965506 | 0.7222 | 0.011275275 | 0.492 | 0.000076866 | 0.8551 | 0.206869361 |
| 46_47_2 | 0.5184 | 0.000000744 | 0.6031 | 0.023513604 | 0.4691 | 0.000004572 | 0.3827 | 0.000000018 | 0.5597 | 0.320900738 |
| 1920 | 1.2452 | 0.003299066 | 1.243 | 0.087285106 | 1.2457 | 0.017226563 | 1.4855 | 0.000071350 | 1.0036 | 0.974575485 |
| 1930 | 1.0812 | 0.307538093 | 1.3317 | 0.025143640 | 0.9697 | 0.746113080 | 1.1421 | 0.204622318 | 1.0197 | 0.860452018 |
| 1940 | 1.0058 | 0.945335997 | 0.9748 | 0.861719370 | 1.0196 | 0.850590593 | 0.9743 | 0.813468318 | 1.0613 | 0.648349664 |
| 1950 | 0.9513 | 0.502001538 | 1.1709 | 0.206001622 | 0.8513 | 0.082698241 | 0.9152 | 0.375428945 | 1.0022 | 0.984202347 |
| 1960 | 1.1583 | 0.057108733 | 1.395 | 0.010927691 | 1.057 | 0.560474063 | 1.2337 | 0.043955491 | 1.0758 | 0.524248947 |
| 1970 | 0.9578 | 0.643303175 | 1.1446 | 0.378369765 | 0.8725 | 0.245028865 | 0.9988 | 0.992853698 | 0.9168 | 0.507700607 |
| 1980 | 1.0021 | 0.977858545 | 0.9619 | 0.755026845 | 1.03 | 0.750053614 | 0.9844 | 0.873982294 | 1.0247 | 0.828652242 |
| 1990 | 1.1062 | 0.319117664 | 1.2287 | 0.222125379 | 1.0458 | 0.723695122 | 1.3085 | 0.048332138 | 0.9201 | 0.581295299 |
| 2000 | 0.8763 | 0.076809556 | 0.9685 | 0.798333786 | 0.8326 | 0.048680477 | 0.9497 | 0.609916205 | 0.7991 | 0.040775013 |
| 2010 | 1.0046 | 0.962662123 | 1.1194 | 0.502770992 | 0.9574 | 0.713884356 | 1.0916 | 0.487640517 | 0.892 | 0.453003680 |
| 2020 | 1.408 | 0.000343675 | 1.4991 | 0.015947535 | 1.3676 | 0.007025025 | 1.7602 | 0.000012970 | 1.1195 | 0.419953028 |
| 2030 | 0.9294 | 0.396867758 | 0.9325 | 0.639674045 | 0.9289 | 0.486493984 | 0.9407 | 0.627115848 | 0.9188 | 0.475201665 |
| 22189_1 | 1.0869 | 0.363449155 | 0.947 | 0.716384489 | 1.1812 | 0.152130948 | 1.1445 | 0.284244277 | 1.0178 | 0.894962462 |
| 22189_2 | 1.4091 | 0.000127834 | 1.2608 | 0.120210779 | 1.5015 | 0.000307060 | 1.6695 | 0.000020755 | 1.1347 | 0.347286690 |
| 6138 | 1.6168 | 0.000000000 | 1.884 | 0.000000658 | 1.4904 | 0.000013167 | 1.7864 | 0.000000006 | 1.4284 | 0.001218962 |
| 6142 | 2.1195 | 0.000000446 | 3.4708 | 0.014147740 | 2.0864 | 0.000002364 | 3.0856 | 0.000000001 | 1.2721 | 0.354828406 |
| 738_1 | 0.7677 | 0.005877835 | 0.8397 | 0.214409027 | 0.7179 | 0.011609442 | 0.7935 | 0.080637868 | 0.7362 | 0.028728770 |
| 738_2 | 0.6227 | 0.000006493 | 0.5114 | 0.000415042 | 0.6732 | 0.002448214 | 0.5162 | 0.000007361 | 0.7867 | 0.107844739 |
| 738_3 | 0.5085 | 0.000000013 | 0.482 | 0.003977395 | 0.5194 | 0.000003371 | 0.5132 | 0.000013714 | 0.4848 | 0.000185023 |

HR, hazard ratio.
